# Energy cost and return for hunting in African wild dogs and cheetahs

**DOI:** 10.1038/ncomms11034

**Published:** 2016-03-29

**Authors:** Tatjana Y. Hubel, Julia P. Myatt, Neil R. Jordan, Oliver P. Dewhirst, J. Weldon McNutt, Alan M. Wilson

**Affiliations:** 1 Structure and Motion Laboratory, Royal Veterinary College, University of London, Hatfield AL97TA, UK; 2 School of Biosciences, University of Birmingham, Edgbaston, Birmingham B15 2TT, UK; 3 Botswana Predator Conservation Trust, Maun, Botswana; 4 Centre for Ecosystem Science, School of Biological, Earth and Environmental Sciences, University of New South Wales, Sydney, New South Wales 2015, Australia; 5 Taronga Conservation Society Australia, Applied Eco-Logic Group, Taronga Western Plains Zoo, Dubbo, New South Wales 2830, Australia

## Abstract

African wild dogs (*Lycaon pictus*) are reported to hunt with energetically costly long chase distances. We used high-resolution GPS and inertial technology to record 1,119 high-speed chases of all members of a pack of six adult African wild dogs in northern Botswana. Dogs performed multiple short, high-speed, mostly unsuccessful chases to capture prey, while cheetahs (*Acinonyx jubatus*) undertook even shorter, higher-speed hunts. We used an energy balance model to show that the energy return from group hunting and feeding substantially outweighs the cost of multiple short chases, which indicates that African wild dogs are more energetically robust than previously believed. Comparison with cheetah illustrates the trade-off between sheer athleticism and high individual kill rate characteristic of cheetahs, and the energetic robustness of frequent opportunistic group hunting and feeding by African wild dogs.

## Abstract (web summary)

The long-distance hunting behaviour of African wild dogs is thought to be energetically costly. Here, Hubel *et al*. show that multiple opportunistic short-distance hunts and group feeding make African wild dogs in mixed woodland savannah energetically robust.

## Graphical abstract

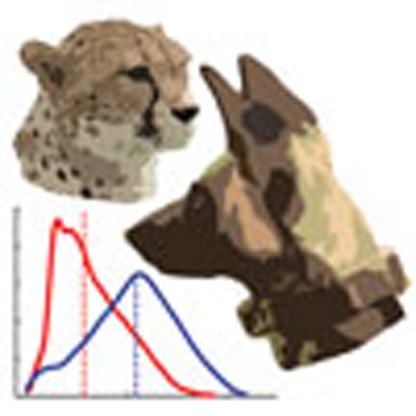

Hunting requires a predator to outperform their prey using a combination of stealth, speed, agility and endurance^1^,^2^,^3^,^4^. Cursorial hunting strategies range from one extreme of transient acceleration, power and speed to the other extreme of persistence and endurance with prey being fatigued to facilitate capture. Cheetahs use high acceleration, speed and manoeuvrability to capture prey in a relatively short chase^4^. Dogs^5^,^6^,^7^,^8^,^9^ and humans^10^ are considered to rely on endurance rather than outright speed and manoeuvrability for success when hunting cursorially. Examples include domestic hounds (*Canis lupus familiaris*) capturing red deer (*Cervus elaphus*) after following it for 3 h over 19 km (1.8 ms^−1^)^5^; and San bushman hunters in the Kalahari Desert running down eland (*Taurotragus oryx*) and greater kudu (*Tragelaphus strepsiceros*) in midday heat over distances of 15–35 km with a mean speed of 1.7–2.8 ms^−1^ (refs 10, 11).

African wild dogs (also referred to as dogs) have been described as the ultimate cooperative persistence predator^6^,^8^,^9^,^12^,^13^,^14^, using sight and scent to hunt individual prey over many kilometres at moderate speed, rather than the outright athleticism of the cheetah^7^,^15^. A perceived high energetic cost of protracted hunting locomotion^16^ compared with ambush predation has led to African wild dogs being attributed with a low margin of energy intake over that required for maintenance^16^ leading to negative energy balance if a large proportion of kills are lost to kleptoparasites^16^.

This description of African wild dogs as persistent hunters, also a recurrent theme in sporting safari literature^17^, is primarily based on observations of the species hunting in open short-grass plains habitats, such as those found in the Serengeti of Tanzania^13^,^18^,^19^,^20^,^21^,^22^. More recent and detailed knowledge of their range and distribution shows the remaining population of the highly endangered African wild dogs live primarily in woodland and woodland savannah habitats^6^,^14^,^16^,^18^ where direct observation of their hunting strategies is considerably more difficult. The survival of African wild dog packs in areas with denser vegetation and often an abundance of medium-sized, easily subdued prey^23^ raises questions about their hunting strategies, energetics and vulnerability to kletoparasitism, particularly in this environment. Similar questions have been raised about cheetahs^24^,^25^,^26^,^27^.

We deployed innovative GPS IMU (inertial measurement unit) collars on all members of a free ranging pack of six adult African wild dogs and recorded their movement at sample rates depending on their activity. Position and speed were recorded at 5-min intervals for moving individuals and hourly fixes for inactive individuals. The 5-min GPS fix rate was increased to 10-s intervals for 2 h daily during the peak hunting period. When individuals were running the collar recorded 300 Hz IMU and 5 Hz GPS data. We also deployed one or more collars in each of 13 other African wild dog packs living in the surrounding area. We analysed previously collected data on five cheetahs in the same study area^4^ to extract the same parameters as for the African wild dogs. Since there is a lack of consistent definition of terms used to describe hunting, we used the following definition in our study; hunting: all locomotion to find food; hunt: the search and pursuit of prey ending in a high-speed run (chase), covering the time and distance from the end of one chase till the end of the next chase by the same individual, regardless of pack activity; chase: runs when maximum stride speeds exceeded 6 ms^−1^ (galloping). We assume all chases to be the end of a hunt. Individual kill rate: the number of chases ending in a kill versus the total number of chases by that individual; pack kill rate: individual kill rate multiplied by number of actively hunting dogs.

Gorman *et al*.^16^ measured metabolic rates in six adult African wild dogs using double-labelled water measurements and found them to be high. They attributed all locomotor energy expenditure to hunting and developed a net rate model balancing energy expenditure with energy gain. They concluded African wild dogs to be highly susceptible to kletoparasitism due to their high hunting costs. Gorman *et al*.'s^16^ conclusion of high hunting costs in African wild dogs has been cited extensively and has been used as a baseline to calculate foraging success and associated survival^28^,^29^,^30^. However, the applicability of Gorman's model has been questioned by Jongeling *et al*.^31^ who reran the model with different parameters to conclude that food availability, intake and energy content are all higher than assumed by Gorman *et al*., and that kleptoparasitism does not have a major detrimental effect. Here we modify the original model to take into account our ability to capture the distance covered in hunts and relate that to the number of dogs participating. Specifically, we used the information gathered about daily ranging distance, hunting distance and speed, as well as kill rate to calculate the energy margin under which our pack operates, assuming medium-sized prey and different levels of kleptoparasitism. We expanded the model to examine whether the return is sufficient to maintain a positive energy balance when supporting dependent offspring, a consideration lacking in the original model and recently identified as an important factor^31^.

Finally, we populate the same model with equivalent data for hunting cheetah to determine the relative energy balance and susceptibility of kleptoparasitism to explore the difference in energetic cost of high-investment and opportunistic hunting strategies.

We determined the energetic cost and return for a pack of African wild dogs hunting antelope and examined whether the return was sufficient to maintain a positive energy balance with different pack sizes and levels of kleptoparasitism. Our results show that short opportunistic hunts of medium-sized prey are relatively cheap, and African wild dogs using this hunting strategy will most likely have a large safety margin with respect to the effects of kleptoparasitism. Comparing the opportunistic hunting style of the African wild dog pack with the same calculations for the high-investment cheetah chases detailed in ref. 4 shows that the higher individual kill rate and gain-to-cost ratio are offset by the group sharing of kills by African wild dogs.

## Results

### Distance covered per day and activity

**Figure 1:**
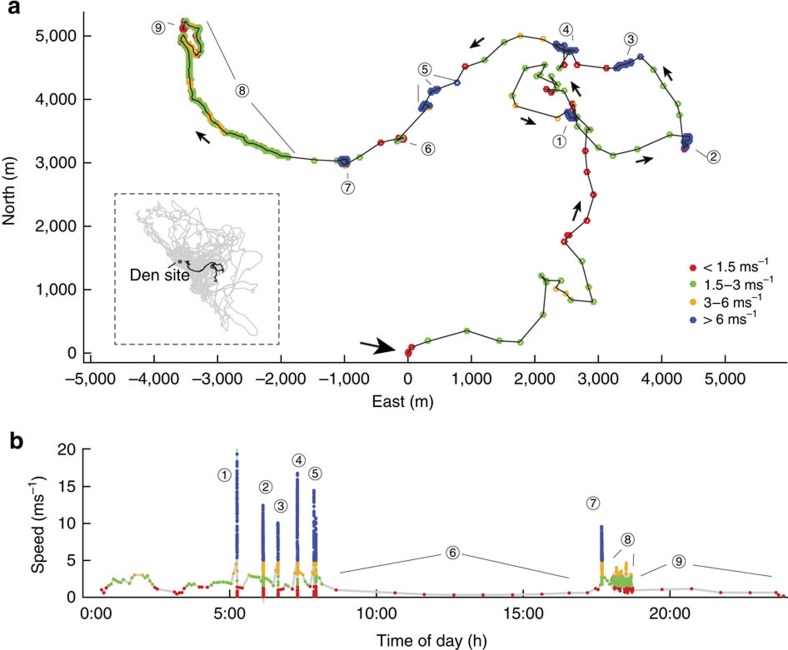
Example day's speed and track for an African wild dog. (**a**) GPS data trace for one individual, dog position at the beginning of the day (0/0), GPS position colour coded based on speed bins, black arrow shows the direction of the movement, inset shows range covered by dogs during 5 months of observation and trace of example day. (**b**) Speed profile at local time for one individual colour coded based on speed bins (legend in **a**). (**a**,**b**) Movement is interspersed by six fast chases (circles 1–5 and 7). The GPS sample rate varies throughout the day and is set to capture high-resolution data when the animal is moving. When the animal is stationary, the sampling interval is once an hour (circles 6 and 9). Between 18:00 and 20:00, it is at 10-s intervals (when moving, circle 8), when high-speed chases are detected, it is increased to 5 Hz (circles 1–5 and 7). At all other times, it is once every 5 min when moving.

**Figure 2:**
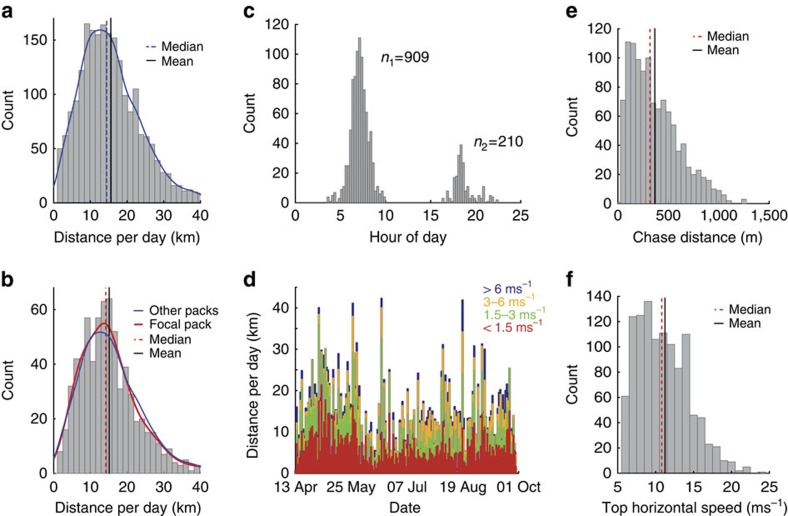
Detailed analysis of African wild dog movement. Histogram of distance travelled per day including kernel density estimate for (**a**) 18 dogs in 13 other packs in the area (*n*=2,226 dog days) and (**b**) for the focal pack (*n*=708 dog days). Abscissa is cutoff at 98th percentile. The kernel density estimate for other packs (blue line) is displayed alongside the estimate for the focal pack (red line) in **b** after being normalized to fit the focal pack sample size. (**c**) Start time for all recorded chases at local time (*n*=1,119). (**d**) Distance travelled per day in kilometres by example dog ‘MJ' colour coded into different speed ranges (walk: 0–1.5 ms^−1^ (red), trot: 1.5–3 ms^−1^ (green), slow gallop: 3–6 ms^−1^ (yellow), fast gallop: >6 ms^−1^ (blue)). (**e**) Total distance covered in each chase (*n*=1,119). (**f**) Top stride speed recorded in each chase (*n*=1,119).

The GPS collars provided position and speed information on individual African wild dogs at sample rates determined by activity and time of day (Fig. 1). The mean distance covered by 18 members of 13 other packs in the area was (±s.d.) 13.78±8.47 km per day (ref 32; Fig. 2a,b; Supplementary Table 2). This is comparable with the study pack, which travelled (±s.d.) 13.20±7.7 km per day (ref. 32; denning female excluded; Supplementary Fig. 1; Supplementary Table 2) and was most active around dawn and dusk^33^ (Fig. 2c). The distance was covered mostly at walk (49% of total distance) and trot (31% of total distance; Fig. 2d)^34^,^35^. The pack travelled up to 42.9 km in 24 h and up to 30 km on consecutive days (Fig. 2d).

### Hunts and chases

We analysed a total of 1,119 chases by the six dogs conducting an average (mean+s.d.) 2.43±0.88 chases per individual per day (ref. 32; Supplementary Table 2). Mean distance covered per chase was (mean±s.d.) 447.3±40.2 m (ref. 32; Fig. 2e; median: 323.8 (Q1: 315.9, Q3: 348.7); Supplementary Table 3; Supplementary Fig. 2). All six individuals achieved a top stride speed of 19 ms^−1^ (ref. 32) at least once (Supplementary Fig. 3) although most runs were considerably slower (Fig. 2f). Chases were interspersed by longer periods of slower locomotion (Fig. 1a). Only 7.4% of total distance covered (Fig. 2d; Supplementary Fig. 1) was faster than 6 ms^−1^ (0.98±1.12 km per day).

**Figure 3:**
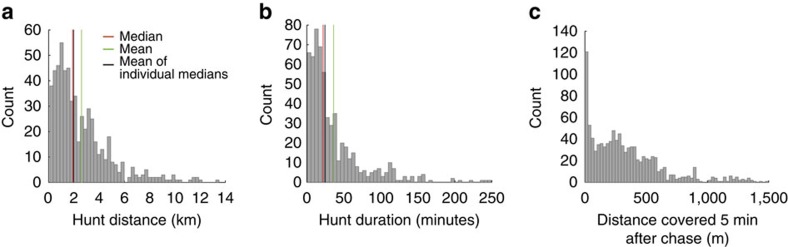
Hunt parameters in African wild dogs. (**a**) Histogram of hunt distance covered from end of a chase to end of next chase by the same individual (the distance of 1,870 m used in the energetic calculation is the mean of the individual dog median values, *n*=1,119). (**b**) Histogram of time elapsed between end of one chase and end of next chase by the same individual (*n*=1,119). (**c**) Histogram of distance covered by individual dogs in 5 min after the end of each of their chases (*n*=1,119, bin width 25 m).

We define the length of a hunt as the total distance (metres) covered to find, approach and chase a prey animal. Hunt distance and duration were determined by calculating the distance and duration between the end of one chase and the end of the next chase of the same dog (Fig. 3a,b; Supplementary Fig. 4). Figure 1 shows five consecutive hunts by one individual in the morning.

A successful hunt (ending in a kill) was defined as a chase after which the animal remained within 50 m of the chase end point for at least 5 min (Fig. 3c). The kill rate expressed on the basis of individual dogs is 0.155 (ref. 32). Applying the same criteria to cheetah data yielded a kill rate of 0.26.

**Figure 4:**
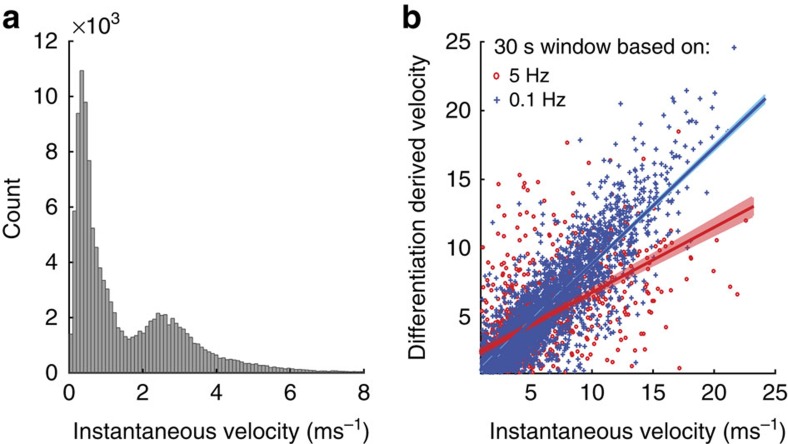
Preferred speed and tortuosity estimates based on instantaneous velocity measures. (**a**) Histogram of instantaneous velocity measures during the daily 2 h of 10 s GPS fixes. (**b**) Differentiation derived velocity versus instantaneous velocity for walking and trotting (10 s GPS fixes, blue, slope 0.84) and running (5 Hz, red, slope 0.47), averaged for 30 s windows. The lower ratio of differentiated to instantaneous velocity for running (5 Hz) shows the higher tortuosity during chases.

African wild dogs showed distinct preferred locomotion speeds (outside of chases) which we attributed to maximum locomotor economy for each gait^34^,^35^. To prove this concept, we display instantaneous velocity, measured by the GPS module, for 10 s GPS fixes (walking and trotting) during the morning hunting period (Fig. 4a). African wild dogs show a preference to move at speeds around (0.35 ms^−1^, walking) and (2.5 ms^−1^, trotting) with the measured trotting speed close to the 2.3 ms^−1^ predicted by the equation of ref. 36 of 1.09 × body mass^0.222^.

To estimate the difference in distance travelled by dogs chasing (hunters) versus non-chasing dogs (followers, presumably following a straighter path), we compared instantaneous GPS-derived velocity values with position-differentiated velocity (both high-speed (5 Hz) and low-speed (10 s) data were averaged over 30 s windows). If travelling in a straight line, then the instantaneous and differentiated velocity will be in agreement (slope=1), while deviations from a straight path are reflected in higher instantaneous velocity and lower slope. Figure 4b shows that while at slow speeds animals do not travel in an exactly straight line (slope=0.84) the tortuosity during chase is considerably higher (slope=0.47). We therefore conclude the distance travelled by a follower to be approximately half that of a hunter during chases.

### Cheetah comparison

**Figure 5:**
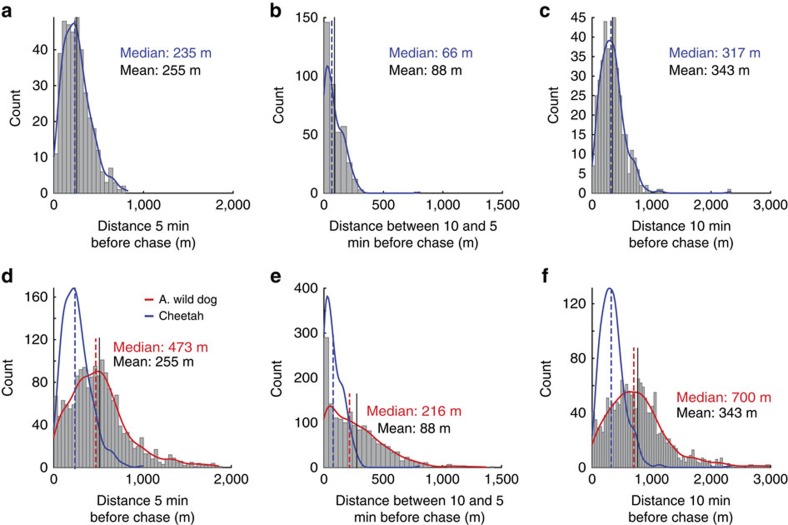
Comparison of African wild dog and cheetah hunt parameters. Histogram and kernel density estimate for cheetah (**a**–**c**; number of cheetahs *N*=5; number of hunts *n*=381) and African wild dog (**d**–**f**; number of dogs *N*=6; number of hunts *n*=1,119; including cheetah kernel density estimate normalized to African wild dog count (blue)). Distance covered 5 min before the start of chase (**a**,**d**) between 5 and 10 min before chase (**b**,**e**) and 10 min before chase (**c**,**f**).

**Figure 6:**
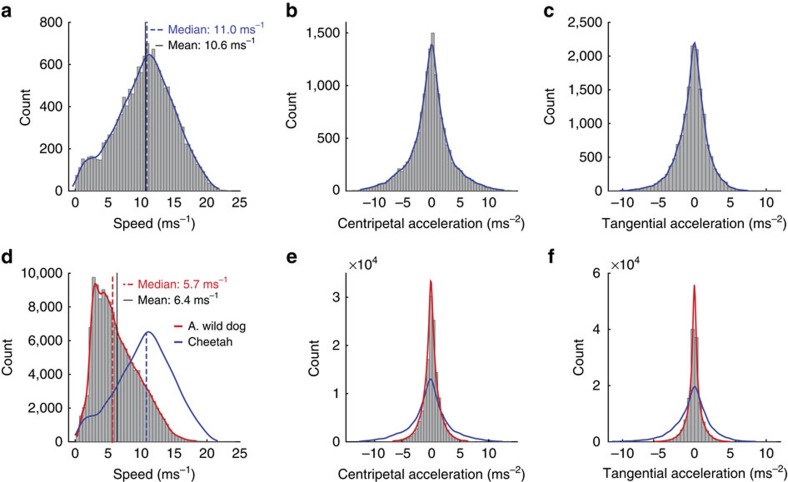
Comparison of African wild dog and cheetah stride parameters. Histogram and kernel density estimate for cheetah (**a**–**c**; number of cheetahs *N*=5; number of strides *n*=15,845) and African wild dog (**d**–**f**; number of dogs *N*=6; number of strides *n*=140,141; including cheetah kernel density estimate normalized to African wild dog count (blue)). Horizontal stride speed (**a**,**d**); centripetal acceleration (**b**,**e**); tangential acceleration (**c**,**f**). African wild dog chases contain about twice as many strides as cheetah chases with a considerably greater number of straight strides at steady speed.

Cheetah and African wild dogs differ in the pre-chase phase as well as in their chase performance. In 10 min preceding a chase, an African wild dog covered 700 m (median) and a cheetah only 317 m (median), reflecting stalking versus coursing (Fig. 5). African wild dog chases contained 87±52 (mean±s.d.) strides while cheetah chases contained only 40±25 (mean±s.d.). African wild dog chases showed much lower peak centripetal acceleration than cheetahs^4^ (7.0 ms^−2^ versus 13.1 ms^−2^) as well as lower tangential accelerations^4^ (8.0 ms^−2^ versus 13.0 ms^−2^) and a proportionally larger number of straight strides at lower, constant speeds (Fig. 6).

### Model of energetic cost and return from hunting

To calculate the energy margin for free ranging African wild dogs, Gorman *et al*.^16^ conducted double-labelled water measurements on the adult members of a pack comprised of six adults, 16 yearlings and 27 pups. They then used a net rate model to balance energy gain with expenditure:


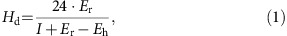


where *H*_d_ is the daily required hunting time, *I* is the prey capture rate (intake rate), and *E*_r_ and *E*_h_ are the energy expenditure rates when resting (basal metabolic rate) and hunting (hunting rates include locomotor and resting costs), respectively. They measured an energy expenditure of 15.3 MJ and derived a resting cost of 4.47 MJ from the literature (20.55 h × 217.5 kJ resting rate). The remaining 10.83 MJ was assumed to be expended during the 3.45 h per day the pack was absent from the den, yielding a cost of 3.14 MJ^−1^ and an intake of 4.43 MJ^−1^ during hunting. The assumption was made that the entire period and energy expenditure was attributable to hunting. They then concluded that a loss of 25% of their prey would increase hunting times to over 12 h a day. The same formula has subsequently been used to calculate predator–prey body size correlations^37^, substituting the direct metabolic measurements with resting^38^ and hunting^39^ costs derived from the literature using estimated average travel speeds (*v*) proportional to body weight (Mb)^38^,^39^, *E*_h_=10.7·Mb^0.684^·*ν*+6.03·Mb^0.684^. For a 25-kg African wild dog, this results in a hunting cost of 1.37 MJ when hunting for 3.45 h or 2.40 MJ when hunting for 6 h as suggested by Carbone^37^. This is in stark contrast to Gorman^16^, who attributes 10.83 MJ of the daily expenditure to hunting of which 10.08 MJ are net locomotion costs (15.3 MJ−24 h × 217.5 kJ). This major discrepancy suggests that either (i) African wild dogs have considerably higher hunting locomotion costs than body size suggests (ii) not all locomotor costs are attributed to hunting or (iii) the energy gained in 3.45 h of hunting exceeded the daily energy expenditure. The neglect of the food intake of dependants (yearlings and pups) in Gorman's calculation has been raised previously^31^.

Here we develop that model as follows. The additional energy consumption during hunting (compared with resting) is largely attributable to locomotion, and the actual cost incurred can be determined from the distance each dog covers at low and at high speed while hunting (and separate from ranging). We determine the distance covered at low and high speed in each hunt along with hunt outcome (kill or no kill) and calculate a cost for actively hunting dogs and following (non-hunting) dogs. We separate the hunting and following dogs, since their energy consumption does differ because the hunters undertake high-speed locomotion and cover a greater overall distance. Hunters also actively contribute to increase the pack's kill rate. We then explored the impact on energy return of pack composition, prey size, hunt distance (which implies prey density), cost of transport (*COT*) estimates and kleptoparasitism. The model development is also displayed in Supplementary Fig. 5.

The Gorman model balances energy expenditure (resting and hunting) with energy gain and takes the form:


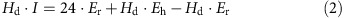


Given that our expenditure calculation is based on distance covered and *COT*, we parameterise the model power as total energy inputs (*E*_In_) in MJ per day and convert to hunting time at the final stage. In addition, our locomotion costs (*E*_Locomotion_=*H*_d_ × *E*_h_−*H*_d_ × *E*_r_) are net locomotion costs, that is, costs in excess of the daily basal metabolic costs (*E*_BMR_=24 h × *E*_r_). Gorman's model therefore takes the form:


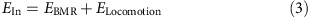


We then distinguish between locomotion costs for hunting (*E*_Hunting_) and non-hunting (*E*_Range_) purpose to take the form:


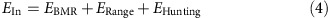


*E*_Range_ and *E*_Hunting_ are net locomotion costs above the resting cost, and are based on the distance travelled during each activity and net metabolic cost of moving a metre (net *COT*, J m^−1^). *COT* is assumed to be lowest and almost independent of gait^35^ when moving at a preferred speed within a gait, but increases, when deviating from preferred speeds^35^,^40^. All costs are calculated in mega Joule (MJ) for a 29 kg dog. Both *E*_In_ and *E*_Hunting_ depend on the number of kills per day (*n*_K_):


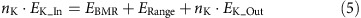


where *E*_K_In_ is the energy intake per kill (depending on prey size, energy value and average daily consumption) and *E*_K_Out_ is the energetic cost per kill:


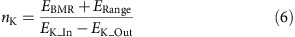


The cost per kill for an individual dog depends on the locomotor cost per hunt (*E*_Hunt_) and the number of hunts an individual has to perform, on average, to make a kill (individual kill rate, *KR*_i_).


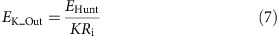


A hunt is subdivided into low-speed (walking and trotting at preferred speed) phases and high-speed chase phases (canter and gallop), and different locomotor costs (*COT*) are attributed to low (preferred) and high speeds^40^.


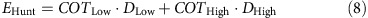


where *D*_Low_ and *D*_High_ are the distances covered at low and high speeds, respectively, and *COT*_Low_ and *COT*_High_ are the corresponding net costs of transport. So these equations give the energy required for a solitary dog to make one kill and relate that to the daily requirements for maintaining energy balance.

### Extending model to account for pack size and composition

The next stage extends the model to account for the fact that the cost per kill depends on pack size and composition in a nonlinear fashion. The number of kills is then represented by equation (6) multiplied by the number of dogs, for fixed costs such as ranging and resting and pack-specific kill gains and costs. The energy balance for the pack is:


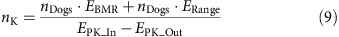


where *n*_Dogs_ is the total number of dogs in pack, *E*_PK_Out_ is the pack's energy expenditure per kill and *E*_PK_In_ is the pack's energy gain per kill.

*E*_PK_In_ depends on number of dogs eating, their average consumption in kg and energy value of content eaten in MJ kg^−1^and has an upper limit based on the prey size.

**Figure 7:**
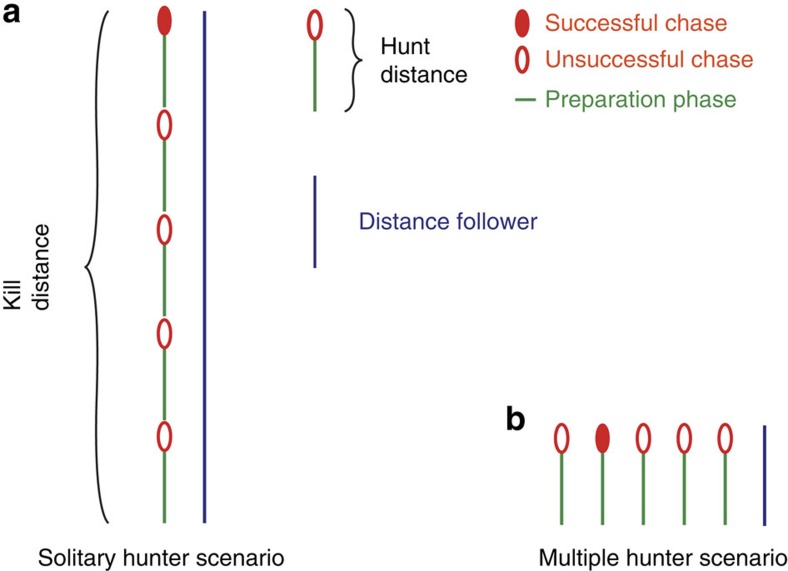
Comparison of solitary versus group hunting scenarios. Solitary hunter (**a**) and multiple hunter (**b**) scenarios assuming a fictional individual kill rate of 0.2. On average, five hunts have to be conducted for one kill. Those hunts can be conducted consecutively by one hunter or simultaneously by five hunters. The kill distance is the sum of all hunt distances and is equal in both scenarios. However, costs (proportional to distance travelled) for individual hunters and for followers is lower for the multiple hunter scenario (**b**).

*E*_PK_Out_ changes with pack composition, and is the sum of locomotion cost associated with the number of dogs actively hunting (*n*_Hunters_) and the locomotion cost incurred by the number of dogs travelling with the hunting party (*n*_Followers_), but not participating in hunting:


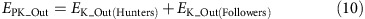


where *E*_K_Out(Hunters)_ is the energy expenditure per kill for all hunters (equation 11) and *E*_K_Out(Followers)_ the cost for all followers (equation 12). The distinction is required to reflect the fact that hunters run at high speed, incurring a higher cost and covering a greater distance (Fig. 4) and increase the pack's kill rate. In a pack such as ours that is comprised entirely of actively hunting members *E*_PK_Out_=*E*_K_Out(Hunters)_. The pack's kill rate (*KR*_p_) increases proportionally with the number of dogs hunting (*KR*_p_=*KR*_i_ × *n*_Hunters_); or in other words, the number of hunts per kill stays the same, but multiple hunts can be conducted simultaneously, as illustrated in Fig. 7, distributing the distance travelled per kill between individuals as well as reducing the time to achieve a kill (assumptions: no cooperation; individuals act as solitary hunters even if chases happen simultaneously, no change in individual kill rate when in a group^32^):


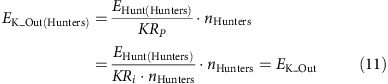


Followers do not contribute to the pack's kill rate or chase frequency, but incur additional locomotor cost:


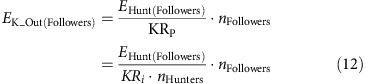


The cost per kill *E*_Hunt_ is higher for hunters than followers due to the followers' lower speeds and reduced tortuosity (Fig. 4b), therefore:


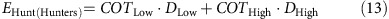



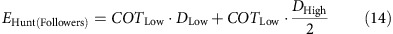


To calculate the daily hunt time (*H*_d_) based on number of kills (*n*_K_), hunt duration (*H*_H_) and pack's kill rate (*KR*_P_):


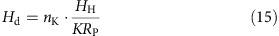


### Basal metabolic cost

To populate equation (9), we calculate *E*_BMR_ at 243.2 kJ h^−1^ for our 29 kg (mean mass of adults in the pack) African wild dogs using the same empirically derived formula for domestic dogs^41^ that was used by Gorman for his 25 kg dogs; *E*_BMR_=24 h × 243.2 kJ h^−1^=5.84 MJ.

### Ranging cost

Not all locomotion is hunting and we assume that dogs move at preferred speeds when ranging (Fig. 4a). *E*_Range_ is the result of ranging distance and *COT*^35^. Having established the use of preferred walking and trotting speeds in our dogs, we use the same *COT* for walking and trotting. A published, speed-independent energetic *COT* for African wild dogs taken from regression line of ref. 39 results in a net cost (*COT*_Low_) of 107 J m^−1^ for 29 kg dogs. Using a ranging distance of 7,176 m per day (median distance travelled per day (11,720 m)–median hunt distance (1,870 m) × chases per day (2.43)); *E*_Range_=7,176 m × 107 J m^−1^=0.77 MJ.

### Kill gain

We calculate the food intake based on the upper end of an average daily consumption of 2.5–3.5 kg (refs 7, 16, 19). Assuming the carcass to contain a median of published energy values of 6 MJ kg^−1^ (refs 16,19), the average maximum energy gained from a kill is 3.5 kg × 6 MJ kg^−1^=21 MJ per dog.

No direct information on prey size and species is available for our data set. However, over 80% of prey captured by African wild dogs in this study area are impala (*Aepyceros melampus*)^42^. Impala calving occurs at the beginning of the wet season^43^ in November. This study ran from April to September, so small calves were not available, leaving a range of impala size between juvenile (5 months growing to 10 months through the study) and male adults, that is, from 20 to 60 kg (ref. 44). For the purpose of this model, we assume an average prey weight of 40 kg (female impala) of which at least 60% is edible^19^, leaving a maximum energy gain of 40 kg × 0.6 × 6 MJ kg^−1^=144 MJ.

For a pack of dogs, the energy gained per kill (*E*_PK_In_) increases linearly with the number of dogs eating until a number of dogs is reached that consume the entire prey. *E*_PK_In_=21 MJ × *n*_Dogs_; limit 144 MJ.

### Kill cost

The cost per kill depends on distance travelled in a hunt, the kill rate, the *COT* and the pack's size and composition. We measured a 0.155 individual kill rate for hunts (Fig. 3c) when treated as solitary event *KR*_i_=0.155. We analysed the hunting energetics data for each dog separately, meaning that values were calculated for each individual and then combined when evaluating the pack's performance and energy balance. We used the mean of individual dog median values to populate the model as a representative measure of typical hunts.

We calculate hunt cost (*E*_Hunt_) based on distance covered and *COT*. While *COT* at preferred speeds is gait independent, it has been argued that for dog-sized animals the *COT* is approximately four times higher than at preferred speeds when manoeuvring, accelerating and chasing at high speeds^40^. We therefore divided the hunt into two phases: the phase to seek out prey, which is covered at low (preferred) speed, and the chase phase that is covered at high speed. Median distance per hunt is 1,870 m (Fig. 3a; Supplementary Table 2), with a median chase distance of 324 m, and the remaining 1,546 m at preferred speeds of walk and trot: *E*_Hunt (Hunter)_=107 J m^−1^ × 1,546 m+(4 × 107 J m^−1^) × 324 m=0.30 MJ (equation 13).

If a dog does not participate in the chase, the distance of the chase is halved due to reduced tortuosity (Fig. 4) and covered at lower speed: *E*_Hunt(Follower)_=107 J m^−1^ × 1,546 m+107 J m^−1^ × (0.5 × 324 m)=0.18 MJ (equation 14).

For a pack entirely composed of hunters (so dogs are hunting simultaneously but not necessarily chasing simultaneously), *E*_PK_Out_=*E*_K_Out_=0.3 MJ/0.155=1.96 MJ (equation 11).

### Pack size and composition

**Figure 8:**
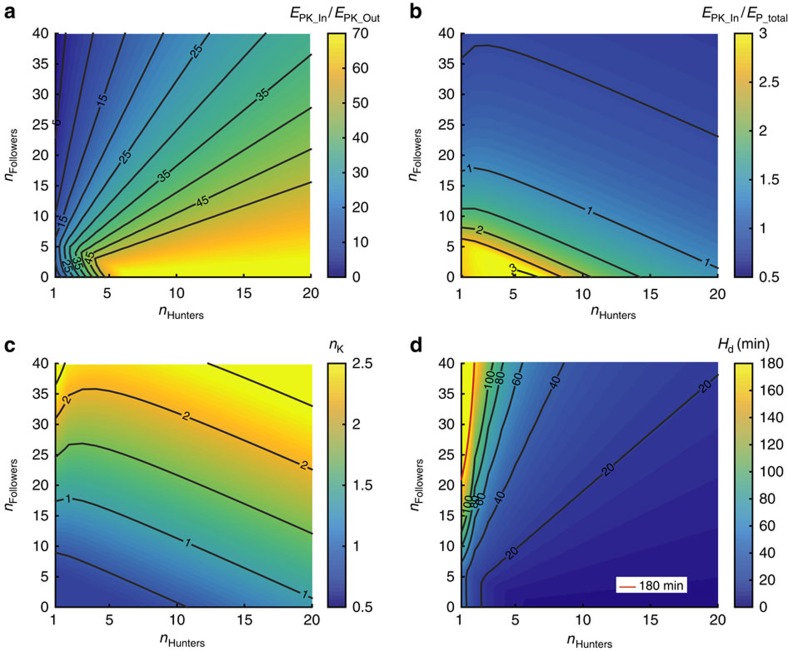
Energy model outcome for African wild dogs. (**a**) Maximum pack energy gain per kill (*E*_PK_In_) divided by pack energy expenditure per kill (*E*_PK_Out_) for different pack sizes and compositions (40 kg impala, 3.5 kg dog stomach capacity). (**b**) Maximum pack energy gain per kill (*E*_PK_In_) divided by total daily pack energy expenditure including one kill (*E*_P_total_) for different pack sizes and compositions (sustainability line at 1). (**c**) Number of kills necessary to fulfil daily energy requirement of pack (basal metabolic cost+ranging cost+*x* times kill cost). (**d**) Total daily hunting time based on number of kills required; based on kill rate and time between hunts (22.58 min), red line indicated 3 h daily hunting boundary.

**Figure 9:**
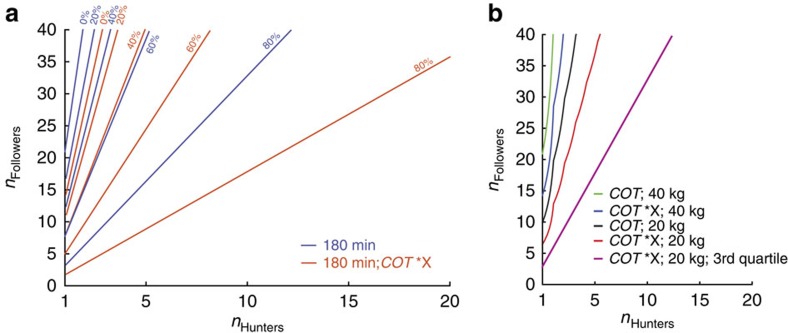
Influence of different model parameters on model outcome. (**a**) Effect of kleptoparasitism and *COT* error analysis. Boundary on sustainable pack composition assuming a 180 min limit on daily hunting time. Red lines indicate boundary (pack composition to the right of the line is sustainable) for different percentages of prey stolen (40 kg, impala). Blue lines indicate sustainability for higher *COT* (*COT*_Low_ × 2.5; *COT*_High_ × 1.5) based on ref. 47. (**b**) Sustainability boundary for 180 min daily hunting time limit calculated for different *COT*^47^, prey size and abundance. Lower prey density simulated using the 3rd quartile for hunt distance (3.38 km) and hunt duration (45.3 min).

We calculated the kill gain to kill cost (*E*_PK_In_/*E*_PK_Out_) ratio for packs of different size and composition (Fig. 8a) and showed that this ratio is very favourable for a large range of pack sizes and compositions. When expressed as the ratio of kill gain to total daily expenditure (*E*_PK_In_/*E*_P_total_; Fig. 8b) it shows that one 40 kg impala can sustain a large number of dogs. If the need arises to hunt again, the cost of making an additional kill (*E*_PK_Out_) is easily balanced energetically. The number of kills necessary to sustain a pack (Fig. 8c) is quite low even for very unfavourable pack compositions of one or two hunters and many followers. However, while the energetic costs are low, total hunting duration may be considerably more critical. Figure 8d shows the total daily hunting time (*H*_d_) required to make the required number of kills rises sharply with a decreasing number of hunting individuals in a pack with many followers. The effects of kleptoparasitism and variation in prey size and *COT* are displayed in Fig. 9a,b.

## Discussion

Accounts and public perceptions of African wild dog hunting are of large packs chasing down large antelope over long distances on the open grass plains. The historical decline in African wild dog numbers has been attributed to a variety of reasons including predation (including humans), habitat loss, diseases, interspecific kletoparasitism and very high hunting costs^16^,^22^,^45^. Our focal pack inhabits an area with significant scrub and woodland, and displays a very different hunting style. Rather than long collaborative pursuits of one large prey, they hunt opportunistically, executing short high-speed chases after one or more medium-sized prey. Calculating locomotor expenditure for this short opportunistic hunting style shows that hunting is energetically rather cheap in comparison with total daily energy expenditure. However, time available for hunting can be a limiting factor due to increased heat and predator encounters or decreased prey availability at different times of the day. Gorman *et al*. estimated a daily hunting and ranging time of 3.45 h for their pack. Figure 8d shows that even when assuming a maximum hunting time of ∼3 h per day (duration of morning hunting period), even a single dog could theoretically support a large number of followers (up to 22). However, due to the nature of our study, we rely on estimates of two factors that could influence the outcome considerably: prey species and size, and *COT*. We changed both of these variables in turn and together, and reran the analysis to evaluate the robustness of our conclusions (Fig. 9b). Following the argument that African wild dogs might prefer juvenile impala and considering it is unlikely that the kill detection methods used will detect small prey such as hare (similar to direct observation, it will be biased towards larger prey that take longer to eat), we assumed an average prey size of 20 kg rather than 40 kg. In addition, studies have shown that *COT* can be influenced by surface properties and could be considerably higher on softer ground^46^,^47^,^48^. The net *COT* for human walking and running in sand has been measured to be 2.1–2.7 (walking) and 1.6 (running) times higher than on a hard surface; long grass would also increase *COT*. We applied a factor of 2.5 and 1.5 to our low- and high-speed *COT*, respectively^47^. Figure 9b shows the effect of reduced prey size, increased *COT* on pack sustainability when assuming a 3-h hunting limit. Despite these most conservative assumptions our pack of six adult hunting dogs could still theoretically support almost 40 followers, showing that energy balance is not as critical in our population as previously described elsewhere. Using the upper quartile for hunt distance and duration to simulate the impact of potentially lower prey density (Fig. 9b), also shows that prey density has a significant effect on the energy balance especially for very small packs; however, despite this, our six-dog pack could theoretically still support up to 16 followers.

The loss of prey (mainly to spotted hyaena (*Crocuta crocuta*)) and its impact on African wild dog population has been discussed extensively^16^,^21^,^30^,^31^,^45^,^49^,^50^. The rate of kletoparasitism differs considerably across ecosystems and has been linked to habitat and hyaena density^49^. In open habitats, kletoparasitism is common and the ability to defend kills against hyaena depends on pack size^21^. So, the assumption that kletoparasitism is very detrimental to maintaining positive energy balance would apply if dogs already operate with a low margin of energy excess in areas without kletoparasitism^16^,^50^. However, our calculations of locomotor costs based on the hunting strategy of short, high-speed chases in our pack do not support this claim. Even when accounting for the cost of dependants, hunting costs are low in comparison with total daily energy expenditure. Calculations on sustainable pack size and compositions for different amounts of prey carcass stolen (Fig. 9a) show the relatively large margin of energy excess in a African wild dog pack of our size, allowing for substantial amounts of carcass being stolen.

If prey density were low (impala, the most common prey, density is ∼15 impala per km^2^ in the study area^51^,^52^), then dogs would have to travel further between chases. In this context, hunting time may exceed the optimum time window and be compromised by heat or light. This effect would be most marked in the hot season rather than the cooler denning season and in smaller packs, and may be exacerbated in the future by warming effects of climate change.

As our pack hunted daily and killed and ate frequently, we chose an average daily consumption of 3.5 kg (refs 7, 16, 19). However, African wild dogs are capable of eating up to 9–11 kg (refs 19, 21), likely when they have not have eaten for several days, which would make them robust to energy deficit during periods of low kill rate. In these situations, a medium-sized prey would feed fewer individuals to the maximum possible energy intake so there would be a benefit in capturing larger prey.

The most critical time for very small packs would be during the denning season when the dominant female, any additional guards, and pups will be fed via regurgitation, effectively reducing an individual's prey intake even if the prey is not completely consumed (dogs hardly ever return to a carcass). However, this might be compensated for by the capacity to eat more than the assumed 3.5 kg.

Loss of kill would have a much higher impact if the energetic cost per kill was high, such as in long endurance hunts of larger prey where many dogs invest substantial energy expenditure in one kill. In this case, larger pack sizes and the ability to defend the kill against hyaena^21^ could be crucial.

Nothing is known about the hunting style of the pack studied in Gorman *et al*. Due to habitat, they assume no loss to hyaenas. They also assume the yearlings to be effective hunters. However, if the yearlings were not yet effective hunters^18^, this would increase the number of dependants, altering the pack composition significantly.

Both African wild dogs and cheetahs thrive in our study area and both hunt mostly impala (>80% of kills for both species^12^,^14^,^42^) but have very different hunting styles. The cheetah stalks and sets up a hunt, while in our study area African wild dogs routinely travel at higher-speed (Fig. 5) flushing prey and then undertaking many opportunistic chases. In our focal pack, individuals chased prey almost twice as often as cheetah did, with chases containing almost twice as many strides^4^. The highest speeds for African wild dogs were 5 ms^−1^ slower than for cheetahs^4^,^53^ and African wild dog peak centripetal acceleration, a measure of maximum turning performance, was almost half that of cheetahs^4^. The proportion of strides at high speeds or high tangential and/or centripetal acceleration were lower in African wild dogs (Fig. 6). It is therefore unsurprising that dogs had a lower individual kill rate than cheetahs (0.155 versus 0.26).

We tested the hypothesis that hunting in African wild dogs has a lower hunt return than cheetahs due to the lower individual kill rate and longer hunts, despite the benefits of group hunting and group feeding, by parameterising the above model with equivalent data for five cheetahs in the study area^4^. We use the *COT* extracted from ref. 40 and a prey size of 40 kg for both the species.

African wild dogs cover double the distance of cheetahs each day (Median distance per day: African wild dog 11.8 km; cheetah 5.6 km). For this calculation, half the cheetah locomotion is taken as searching out and hunting (similar to our focal pack of African wild dogs), while the median distance covered during chasing is 139 m. This yields a distance of (2,800−139)/0.26=10,235 m at low speed and 535 m at high speed per kill. Mean cheetah mass was 50 kg (ref. 4) yielding 154 J m^−1^ for *COT*_Low_ when moving slowly and 581 J m^−1^ for chase *COT*_High_ when moving quickly^40^; this gives a kill cost of *E*_K_Out_=1.89 MJ (solitary African wild dog or pack with no dependants was *E*_K_Out_=*E*_PK_Out_=1.94 MJ). Cheetahs (50 kg) are larger than African wild dogs and eat approximately the same proportionally^24^,^27^, that is, 7 kg and *E*_K_In_=42 MJ. This gives a kill gain/kill cost ratio (*E*_K_In_/*E*_K_Out_) for a solitary hunting cheetah of 22 times locomotor cost—about double the 11 times locomotor cost for a solitary dog. So, a solitary cheetah is, in energetic terms, a more efficient hunter than a solitary African wild dog. However, hunting in a pack can boost the African wild dog pack's kill gain/kill cost ratio (*E*_PK_In_*/E*_PK_Out_) to up to 73, almost triple that of a solitary living cheetah (reduced to double when using^47^ adjusted *COT*). Coalitions of cheetah occur and they will incur similar costs and still feed to satiation so kill gain to kill cost ratio in cheetah coalitions will be similar to those in dog packs. The relatively low hunting costs of both predators suggest neither to be very susceptible to kleptoparasitism from an energetics point of view; a conclusion in agreement with recent studies about cheetah hunting^27^.

The African wild dog pack captured prey by performing short, high-speed chases, while hunting using relatively efficient gaits. Using an energy balance model accounting for kill rate, hunt distance, group composition (hunters and followers) and an estimated prey intake, our calculation of energy return from hunting opportunistically, using short high-speed chases, revealed considerably lower hunting costs than previously suggested. However, while hunting after the loss of kill might be energetically rather cheap, the pack might be compromised by the time window available for efficient hunting, especially in the light of higher temperatures due to climate change and low prey densities with habitat fragmentation. Prey size and consequently hunting style will have a considerable effect on the impact of kleptoparasitism, with losses being harder to overcome when using sophisticated endurance strategies where several animals invest highly in the same hunt.

Comparison of African wild dog and cheetah hunting reveals that while cheetah hunts are energetically more expensive, their higher individual kill rate yields a higher kill gain to kill cost ratio for solitary animals, which is ultimately more than balanced in African wild dogs by the sharing of kills. This illustrates the trade-off between the sheer athleticism^54^ and high individual kill rate characteristic of cheetahs, and the physiology and broad ranging of opportunistically hunting African wild dog packs that rely on sharing kills.

## Methods

### Animals

The packs in this study were located in the Okavango Delta region of Northern Botswana and are part of an ongoing study by Botswana Predator Conservation Trust (http://www.bpctrust.org). Every member of a pack of six adult dogs (‘focal pack') was collared. The pack consisted of a dominant male (‘Kobe') and dominant female (‘Timbuktu'), two subdominant males (‘MJ', ‘Scorpion') and two subdominant females (‘Accra', ‘Kigali'). Data collection on all pack members started on 13 April 2012 and continued over the following 5–7 months, with one collar failing on 27 May. The collar was replaced, but the failure resulted in a lack of data for one dog (‘Accra') over a period of 22 days. This time period was removed from our analysis. Collar removal started at the end of August 2012. One dog (‘Kobe'), the dominant male, died on 27 June. The data from the dominant female (‘Timbuktu') show a period of low activity when she remained at the den with pups. Distance travelled per day was calculated excluding the denning female Timbuktu.

The dogs were immobilized by free darting from a vehicle using xylazine (55 mg), ketamine (50 mg) and atropine (1.1–1.2 mg), and reversed after 45–60 min with yohimbine (4 mg) or atipamezole (5.5 mg). While sedated, anatomic measurements including limb lengths, limb and body girths, and body mass were recorded (Supplementary Table 1). Collar data were retrieved via radio link to a ground vehicle every few weeks.

This work was approved by RVC Ethics & Welfare Committee.

Northern Botswana holds part of one of the largest African wild dog populations. The common kleptoparasites of African wild dogs are spotted hyaenas and lions (*Panthera leo*).

### Comparison with other packs

To demonstrate that our focal pack is representative of all the packs in the area, we used high-resolution GPS collar data from 18 subdominant individuals from 13 different packs in the area. The collars worn by African wild dogs outside the focal pack were either the same as or an earlier version of the collars used on the focal pack. Outside the focal pack data were recorded at 1-h intervals when dogs were resting and at 5- or 10-min intervals when they were moving. Only four collars were allowed to go into ‘run state' for a limited trial period not exceeding a total of 2 months (see collar below). Data were collected for time slots of different duration (21–409 days) between November 2011 and October 2014.

Distance travelled per day and run parameters (maximum stride speed, centripetal (turning) acceleration and tangential (fore-aft) acceleration and deceleration, duration, distance and mean absolute heading rate) were compared using the mean of the individual medians in the focal group and the group containing all individuals from other packs. We used a two-sample *F*-test to check for equal variance and subsequently a two-sample *t*-test or Welch's test; *p* values were adjusted using Bonferroni correction.

We found no significant difference (Welch's test, *p*=0.09) between the mean of the medians of the distance travelled per day by individual dogs of the focal pack and the mean of medians of the other dogs (Supplementary Table 2).

Equally when we compare run parameters (maximum stride speed, tangential acceleration/deceleration, centripetal acceleration, mean absolute heading rate, duration and distance) of our focal pack to four individuals in different packs, only one, mean absolute heading rate, came out to be significantly different (Supplementary Table 3).

### Comparison with cheetahs

We reanalysed previously collected and published GPS/IMU data^4^ from five wild cheetahs (three female and two male) using the same methods as described here to compare locomotor performance of cheetah and African wild dog. The cheetah data were collected in the same study area (in and around the Moremi game reserve, Okavango Delta, Botswana) between July 2011 and August 2013. Data collection continued after publication^4^ and we added the new data into our analysis, bringing the total number of runs analysed from 367 to 488, including 468 chases.

### Collar design and data recording

Power consumption poses a major challenge in the design of a wildlife tracking collar. To fulfil the demands of sufficient data rate during periods of high animal activity and average low-energy consumption, we used collars designed in-house and previously used successfully on cheetahs^4^. The collars use in-built solar cells on the top housing and careful management of the GPS sample rate for power conservation. The mass of the mark two collars was ∼340 g. Dropoff units (Sirtrack; 70 g) were used to release two collars at the end of the study. Other collars were removed following immobilization.

The collar was controlled by a low-power MSP430 16-bit microcontroller (Texas Instruments Inc., TX, USA), running custom software written in the ‘C' programming language. A 2-GB micro-SD flash memory card (Sandisk, CA, USA) was used for on-board data storage.

The collar provides GPS position and instantaneous velocity data as well as three-axis-specific force and rotation rate data. GPS position and velocity were obtained from an LEA-6T GPS module (u-Blox AG). An MMA7331 three-axis accelerometer module (Freescale Semiconductor) provided specific force with a ±12 *g* range. The roll and pitch rotation rate were measured by a dual-axis gyroscope (ST Microelectronics), and yaw rotation rate by a single-axis gyroscope (ST Microelectronics), both set to the 2,000° s^−1^ range. Sensor outputs were filtered by simple single-pole analogue filters (100 Hz knee), and then sampled by the microcontroller at 300 (accelerometers) or 100 (gyroscopes) samples per second. Data download from the collar were via a 2.4-GHz chirp-spread-spectrum communication module (Nanotron Technologies Gmbh). Power was provided by two batteries: a 900-mAh lithium–polymer rechargeable battery (Active Robots), charged by a solar cell array consisting of 10 monocrystalline silicon solar cells (Ixys Koria), and a 13-Ah lithium thionyl chloride battery (Saft). The microcontroller measured both battery voltages and the charge current from the solar cell array, and switched the collar electrical load between batteries depending on the battery state.

To manage power consumption effectively, the collar was programmed to switch dynamically between four different operating ‘states' (Supplementary Fig. 6). The state depended on the time of the day and the animal activity level (measured using the accelerometers). The different states enabled power rationing between average power consumption on the one hand, and quantity and resolution of data on the other. Multiple software updates were installed on the collars (remotely) during the research period to improve performance and capture as many hunts as possible. The default state (‘alert state') provided GPS positions every hour, and allowed the transition into ‘mooch state' with 5-min fixes when the animal was deemed active, based on periodic specific force measurements (measurement taken for 10 s at 30 Hz every minute). Initially, the collar was set to ‘ready state' when the animal was moving between local times of 18:00 and 20:00, since previous work suggested that most hunting occurs around dawn and dusk^33^. In ‘ready state', GPS positions and speeds were recorded every 5 s, if the animal was deemed to be active. A transition occurred from ‘ready' state to ‘run state' if fore-aft accelerometer data exceeded a threshold equivalent to galloping in three consecutive peaks, and the run was defined as valid and stored if five further peaks were detected. In ‘ready state', accelerometer data were recorded into a circular buffer at 100 Hz, the buffer storing the latest 3 s of data. This prebuffering allowed open-loop inertial navigation back to the beginning of the run. However, it was later deemed that an extended time allowed for entering ‘run state' was more beneficial than the prebuffering of data. Prebuffering was abolished on 26 April 2012; this resulted in the loss of the first one or two strides at the beginning of the run. From then on, the collar was allowed to enter ‘run state' directly from ‘mooch state' during preselected ‘times of interests' between 4:00–10:00 and 17:00–22:00 local time. During the ‘times of interest', GPS data were recorded every 5 min (the same as during normal ‘mooch state'), but sample rates were increased to every 10 s for a 2-h window within the ‘times of interest' to get a more accurate account of position during times when most hunts were expected to happen based on initial data observations. Initially, this time was chosen to be between 18:00 and 20:00, and later changed to between 06:00 and 08:00 local time.

### Signal processing

GPS data with horizontal position accuracy above 8 m were removed for all calculations.

In the ‘run state', the power management features used gave different sampling rates for accelerometer (300 Hz) and gyro (100 Hz). GPS position (5 Hz) and instantaneous velocity (5 Hz) were usually (but not always) available within 1 s after entering the ‘run state' but often not accurate until 4–6 s later (Supplementary Fig. 7c–f).

To reduce noise, improve precision and increase temporal resolution in the position and velocity data, GPS and IMU measurements were fused as previously described^4^ using a 12-state extended Kalman filter^55^ followed by a Rauch–Tung–Striebel smoother^56^ written in MATLAB (The Mathworks Inc., MA, USA) (Supplementary Fig. 7a,b).

### Definition of locomotion

There is no global definition of the terms hunting, hunt or chases, and in the context of this study, we define the terms as followed: hunting is all locomotion in the pursuit of food and encompasses multiple (mostly unsuccessful) hunts. A hunt is the locomotion in search (slow speed) and pursuit of a prey individual ending in a high-speed run (chase). We realised that some terms used might require a more extensive explanation due to the two-level analysis carried out to look at individual and pack performance. Terms such as ‘hunt', for example, can be applied to an individual or the pack. At the pack level, it is often defined as the time from the end of one group chase to the end of the next group chase (group hunt). Since not all individuals necessarily participate in a group chase, we defined hunt on an individual basis, encompassing the time and distance from the end of one chase to the end of the next chase by the same individual. A hunt encompasses a slow-speed (search) and a high-speed (chase) phase. Hunters: individuals actively hunting i.e. chasing and killing prey; Followers: individuals accompanying hunters, but not actively pursuing prey (pups and yearlings up to a certain age); Ranging: all non-hunting locomotion such as border patrol or return to the den; Foraging success: Energy gain per day.

### Data analysis

The recording at high-sample rate was triggered by the IMU and continued as long as the horizontal acceleration threshold was exceeded within a 5-s window. Overrun times between 5 and 20 s were implemented depending on the software update. Recordings at 5 Hz were restricted to 87 s and runs exceeding this time, while still showing speeds above 3 ms^−1^ were reconstructed based on 10-s data. We were unable to reconstruct the ending of 5.7% of the runs and assigned an ending randomly chosen out of the pool of reconstructed endings, assuming the distribution is representative for all runs exceeding 87 s. Eighty per cent of the runs lasted <87 s and only a few (2.4%) lasted significantly longer. The difference in median distances covered per run between reconstructed and non-reconstructed data was 2.7%.

Recorded activity lasting <5 s and never exceeding 3 ms^−1^ (instantaneous GPS velocity) were excluded from the analysis leaving a total of 2,026 runs to be analysed; 69 runs failed to produce converged Kalman-filtered results (speed going towards infinity) and were removed. Sufficient strides (at least three per run) were successfully extracted from 1,641 runs. In 4% of the cases (65 runs), a second run was triggered within 30 s of the first ending, and the two recordings were classified as a single run. The two runs were combined by linear interpolation of position and hence speed to fill the gap between them. A total of 1,551 runs (140,141 strides) contained at least one stride whose average speed exceeded 3 ms^−1^ (a speed determined to be slow canter). Runs exceeding a 6 ms^−1^ (galloping) stride speed threshold were classed as chases. We recorded 1,119 valid chases.

### Individual kill rate

Individual chases were automatically classified as successful (ending in kill) if the animal remained for at least 5 min within a 50-m radius of the end of the chase. Automatically determined kills were validated by animating chases and observing pack behaviour such as converging to a supposed kill site and remaining there for at least 5 min (examples in ref. 32 and Supplementary Movie 1).

### Daily distance travelled

Data collected under the different collar states were combined onto a single timeline to determine distance covered per day. Mean speed of each dog when moving slowly was taken as the straight-line distance/time between 5-min GPS fixes so is an underestimate if a tortuous route was followed.

### Calculation of speed and stride frequency

All data analysis was carried out using MATLAB. Fore-aft acceleration was used to determine stride peak times and stride frequency. A band-pass Butterworth filter (4th order) was applied with cutoff frequencies of 1 and 8 Hz, and assuming a maximum stride frequency of 3 Hz, a peak detection function was used to detect peaks with a minimum duration of 0.33 s between peaks and a minimum peak height of 0.5 g. Maximum horizontal stride speed was derived from the Kalman-filtered and smoothed velocity averaged over strides.

### Tangential acceleration, change of heading and centripetal acceleration over stride

Mid-stride times were used to calculate tangential (fore-aft) acceleration, centripetal (turning) acceleration and change in heading between strides. The displacement vectors between consecutive strides were then calculated:


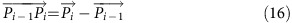


and


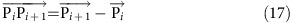


Where 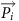 is the two-dimensional position at sample/stride *i*.

Change of heading (Δ*θ*_*i*_) was calculated from the angle between the two vectors:


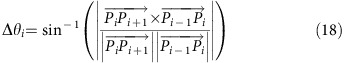


Angular velocity (*ω*_*i*_) was derived by dividing the change of heading by the time between mid-stride positions Δ*T*:


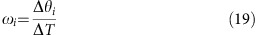


The tangential or fore-aft acceleration (*a*_t,*i*_) and centripetal acceleration (*a*_c,*i*_) were then computed from mid-stride speeds *v*_*i*_:


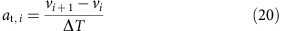



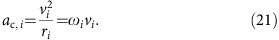


To reduce outliers, tangential and centripetal acceleration were based on weighted stride speed and weighted heading rate taking the stride before and after into account in an approach described in ref. 4 and the companion cooperation paper.

Negative values for tangential acceleration indicate deceleration. Positive and negative values for centripetal acceleration indicate right (+) and left (−) turns. Positive and negative centripetal acceleration values are presented separately to show if there was a preference for left-hand or right-hand turns.

### Run distance

Distances covered within individual runs were calculated by integration of the stride-averaged horizontal speeds over the duration of the run.

Total hunt distance was based on chase distance and the distance covered at lower speeds between the end of one chase to the end of the next chase during a morning and evening hunting session of 5 h each.

### Maximum speed reliability

The maximum stride speed of 19 ms^−1^ was reported for the following reasons: (1) all individuals achieved this speed at least once, (2) 19.4 ms^−1^ is the 99th percentile from maximum stride speeds from all runs. (3) Using only maximum speeds from runs above 6 ms^−1^ the 99th percentile is 20.0 ms^−1^, taking the s.d. of 0.3 ms^−1^ for Kalman-filtered speeds (Supplementary Fig. 7b) and considering a maximum speed measurement error of three s.d.'s gives a maximum speed of 19 ms^−1^.

### Preferred speeds

African wild dogs travel at distinct preferred speeds while ranging and hunting (outside of chases). To prove this concept, we displayed instantaneous velocity measures by the GPS module for 10-s GPS fixes (walking and trotting) during the morning hunting period. African wild dogs show a preference to move at speeds around 0.35 ms^−1^ (walking) and 2.5 ms^−1^ (trotting), with the measured trotting speed close to the 2.3 ms^−1^ predicted by the equation in ref. 36 of 1.09 × body mass^0.222^.

### Path travelled by follower

As all members of our pack were active hunters, our prediction of the behaviour of followers is partly based on direct observation in other packs. We assume that hunters and followers travel approximately the same distance during the slow phase of the hunt since they travel together. During the chase, we used the instantaneous velocity to predict the length of the path travelled by a dog following versus the one chasing. The dog following would not have gone into 5 Hz GPS update chase mode due to the low speed, making it impossible to determine whether it follows the same path as the chasing dog or takes a more direct route. The difference in sample rate between running and non-running dogs prevented a direct comparison of distance travelled; however, the extent of the paths' tortuosity can be estimated by comparing instantaneous GPS-derived velocity values with position-differentiated velocity. For this, both high-speed (5 Hz) and low-speed (10 s) data were averaged over 30-s windows. If dogs travelled in a straight line, then the instantaneous and differentiated velocity would be in agreement (on average). If dogs deviated more from a straight path, then instantaneous speed would tend to be higher than that predicted from distance covered over the 30-s period. We selected times when chases occurred and extracted the data from chasing dogs (5 Hz) as well as followers (10 s; Fig. 4b). The data were noisy due to GPS velocity error (Supplementary Fig. 7b), speed changes throughout stride and over 30 s, and variations in paths followed.

## Additional information

**How to cite this article**: Hubel, T. Y. *et al*. Energy cost and return for hunting in African wild dogs and Cheetahs. *Nat. Commun.* 7:11034 doi: 10.1038/ncomms11034 (2016).

## Supplementary Material

Supplementary Information Supplementary Figures 1-7 and Supplementary Tables 1-3.
