# Supplementary material for: Energy cost and return for hunting in African wild dogs and cheetahs
Source: Nat Commun. 2016 Mar 29;7:11034. doi: 10.1038/ncomms11034 (PMC4820543; doi:10.1038/ncomms11034)
Supplement: Supplementary Information — Supplementary Figures 1-7 and Supplementary Tables 1-3. [file ncomms11034-s1.pdf]

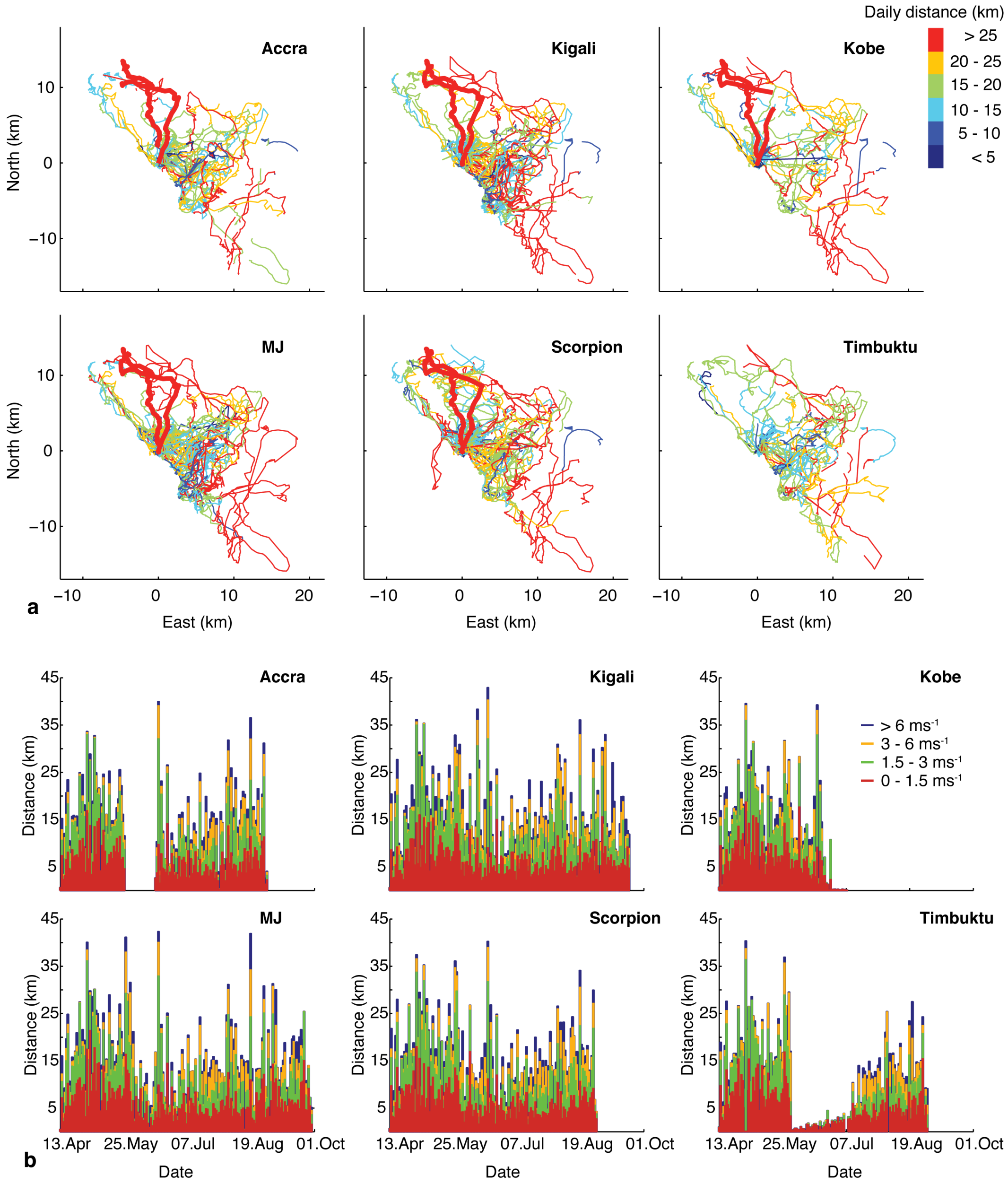

Supplementary Fig. 1:GPS position and distance travelled during collar deployment time. (a) GPS position for all individual dogs colour-coded by distance travelled per day, with den site as origin. Thick line indicates path taken on the 18/6/2012 the day with the greatest distance (42.9 km) travelled. Here we display all data recorded for the individual dogs; however, since data collection period varied between individuals only a subset was used in the analysis (Methods). (b) Distance in kilometres travelled per day by individual dogs at different speed ranges. The gap in Accra's data was due to collar failure while the decreased distance travelled by Timbuktu was due to her remaining at the den site during early denning. Kobe died on June 27th 2012. All animals were collared on April 13th 2012 but the recording time varied. We only used times when all living members of the pack were wearing active collars for our analysis.

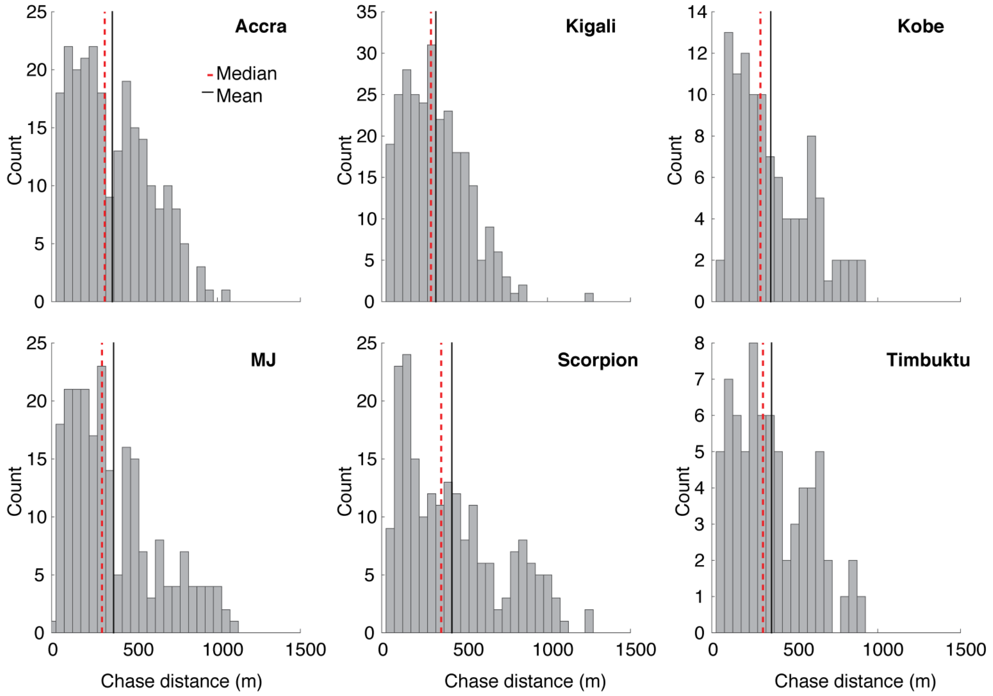

Supplementary Fig. 2: Histograms of distance covered in each chase (run> 6ms^{-1}) for each individual.

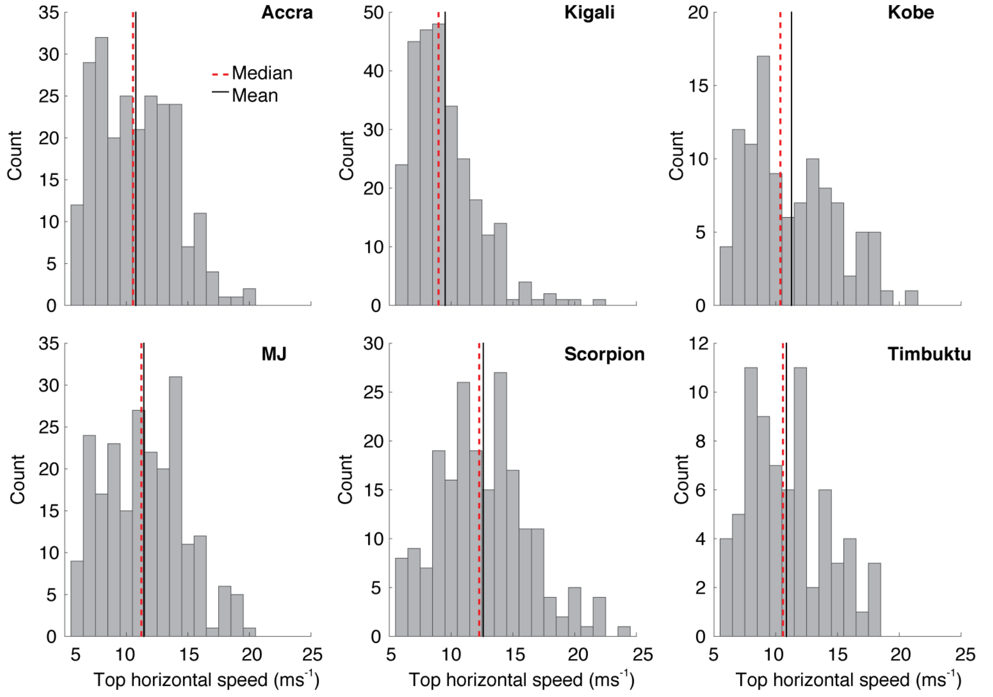

Supplementary Fig. 3: Histograms of top speed in each chase (run> 6ms^{-1}) for each individual.

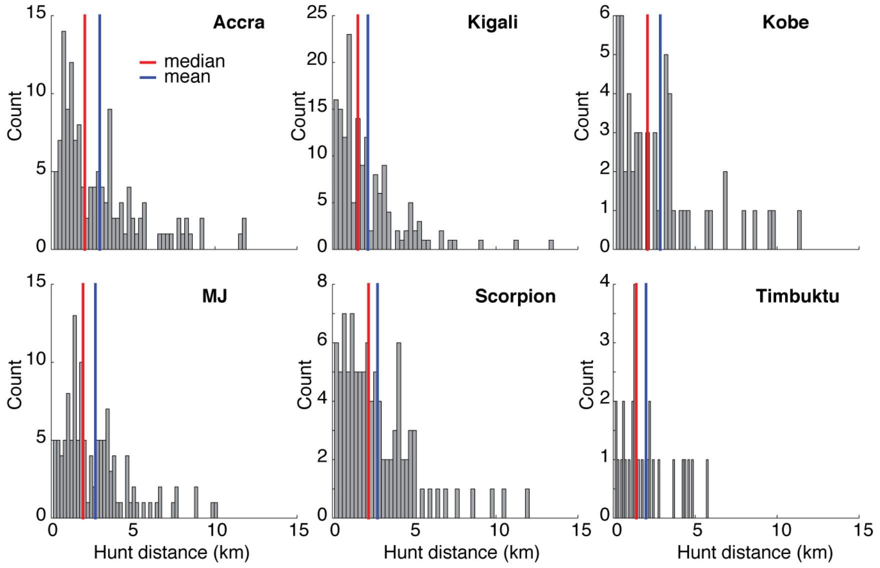

Supplementary Fig. 4: Histogram of distance covered from start of one run to start of next run, based on GPS positions (GPS speed > 5ms for 0.4 of a second to be considered a run) for individual dogs. The distance of 1870 m used in the energetic calculation is the mean of the individual medians.

Gorman: $H_d = 24*E_r / (I+E_r - E_h)$

$$H_d*I = 24*E_r + H_d*E_h - H_d*E_r$$

$$E_{In} = E_{BMR} + E_{Locomotion}$$

$$E_{In} = E_{BMR} + E_{Range} + E_{Hunting}$$

$$n_K*E_{K\_In} = E_{BMR} + E_{Range} + n_K*E_{K\_Out}$$

Hubel: $n_K = (E_{BMR}+E_{Range}) / (E_{K\_in} - E_{K\_Out})$

$$H_d = n_K*H_H/KR_i$$

$$E_{Range} = COT_{Low}*D_{Range}$$

$$E_{K\_Out} = E_{Hunt}/KR_i$$

$$E_{Hunt} = COT_{Low}*D_{Low} + COT_{High}*D_{High}$$

$$E_{K\_In} = 3.5kg*6MJ/kg$$

$$E_{BMR} = 24*217.5kJ/h$$

Gorman et al (Power)

H_d: daily hunting hours

E_h: hunting rate

E_r : resting rate == ***BMR***

I : intake rate

Hubel et al (Energy)

COT_{Low}: net cost of transport at low speed

COT_{High}: net cost of transport at high speed

D_{High}: hunt distance at high speed (chase)

D_{low}: hunt distance at low speed

D_{Range}: non-hunting distance

E_{BMR} : daily basal metabolic expenditure

E_{Hunt}: energy expenditure per hunt (MJ)

$E_{Hunting}$: net expenditure for hunting locomotion (MJ)

E_{K_In}: energy gain per kill (MJ)

E_{K_Out}: energy expenditure per kill (MJ)

E_{In} : total daily intake (MJ)

$E_{Locomotion}$: net locomotor expenditure

E_{Range}: expenditure for non-hunting locomotion (MJ)

H_H: duration of hunt

n_K: number of kills per day

IR_i: individual killrate

Pack:

$$n_K = (n_{Dogs}*E_{BMR} + n_{Dogs}*E_{Range}) / (E_{PK\_in} - E_{PK\_Out})$$

$$H_d = n_K*H_H/KR_P$$

$$n_{Dogs} = n_{Hunters} + n_{Followers}$$

$$KR_P = KR*n_{Hunters}$$

$$E_{PK\_Out} = E_{K\_Out\ (Hunters)} + E_{K\_Out\ (Followers)}$$

$$E_{K\_Out\ (Hunters)} = (E_{Hunt\ (Hunters)}/KR_P)*n_{Hunters}$$

$$E_{K\_Out\ (Followers)} = (E_{Hunt\ (Followers)}/KR_P)*n_{Followers}$$

$$E_{Hunt\ (Hunters)} = COT_{Low}*D_{Low} + COT_{High}*D_{High}$$

$$E_{Hunt\ (Followers)} = COT_{Low}*D_{Low} + COT_{Low}*(D_{High}/2)$$

$$E_{PK\_In} = n_{Dogs}*3.5kg*6MJ/kg$$

$$E_{PK\_In\_max} = 40kg*6MJ/kg*0.6$$

Pack

$E_{Hunt\ (Hunters)}$: energy expenditure per hunt (MJ) for hunters (= E_{Hunt})

$E_{Hunt\ (Followers)}$: energy expenditure per hunt (MJ) for followers

E_{PK_In}: pack energy gain per kill (MJ)

- $E_{PK\_In\_max}$: max energy in prey

E_{PK_Out}: pack energy expenditure per kill (MJ)

- $E_{K\_Out\ (Hunters)}$: energy expenditure per kill for all hunters
- $E_{K\_Out\ (Followers)}$: energy expenditure per kill for all followers

n_{Dogs} : total number of dogs

- $n_{Hunters}$: number of hunting dogs
- $n_{Followers}$: number of dependants travelling with pack

IR_p: pack kill rate

Supplementary Fig. 5: Model development from Gorman et al to Hubel et al. Due to the parameters measured in the current study it was necessary to convert the model from power

input (rates) to energy input and locomotor costs were net costs in addition to the daily basal metabolic costs.

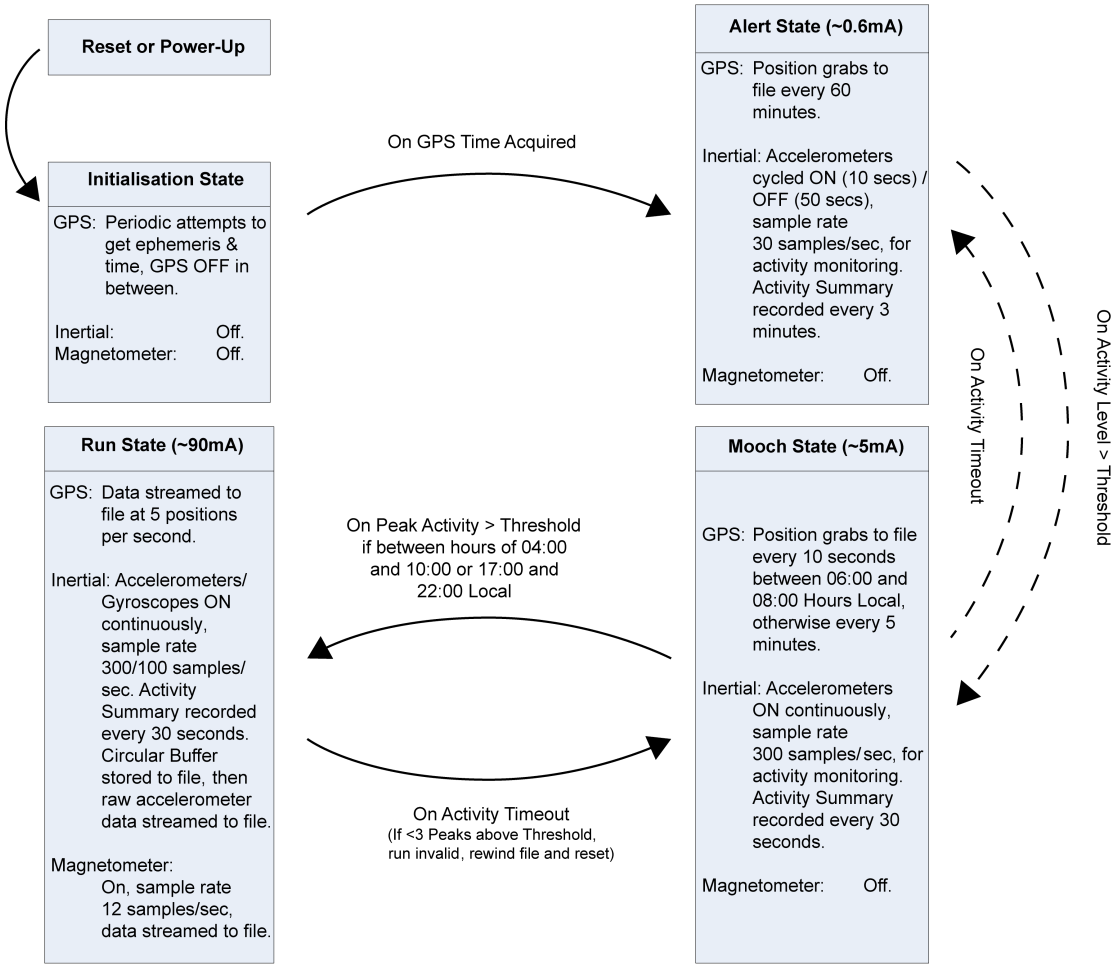

Supplementary Fig. 6: Wildlife collar state and transition diagram as used during the majority of data collection time 21 May – 31 Aug. 2012. Collar settings were optimised during the first few weeks after deployment on 13 April 2012, most notable was the eradication of pre-buffering in favour of an extending time period when collars were allowed to go into chase state on 26. April 2012 and the switch from mooch state with position grabs every 10 seconds from 18:00-20:00 to 6:00-8:00 on 21 May 2012.

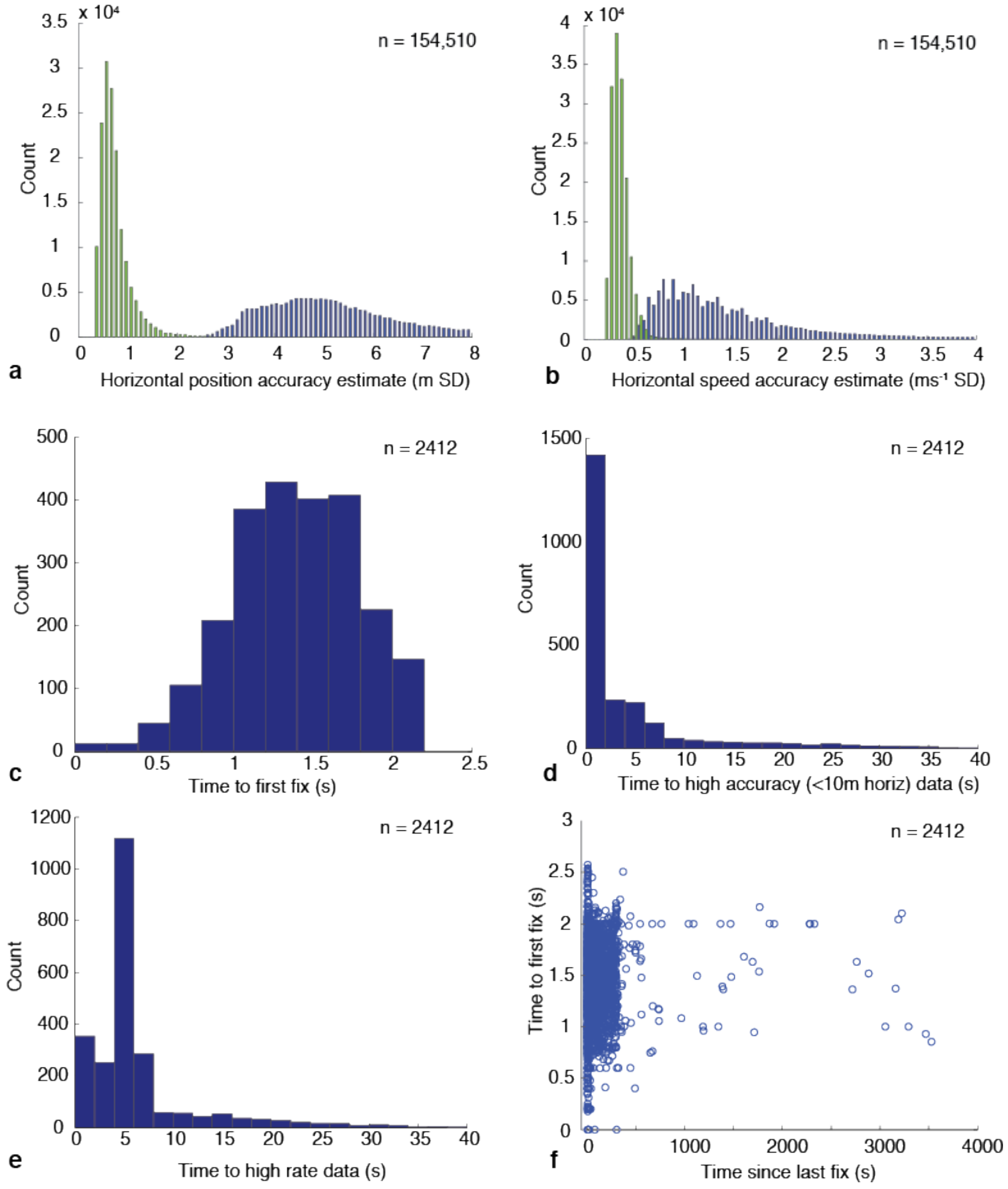

Supplementary Fig. 7: Summary of GPS performance in the field during the study. (a) Horizontal position accuracy standard deviation estimate, (blue) from the GPS module for the stand alone GPS data, (green) from the Kalman smoother once GPS and IMU data have been fused. The estimation algorithm inherently gives error covariances of each parameter for each sample based on measurement error calculated for each fix by the GPS module and IMU errors from lab testing. GPS horizontal position error (median stridewise SD) was reduced by data fusion from 5.84±2.71 m (pure GPS data) to 0.72±0.36 m in the smoothed solution. (b) Equivalent plot to (a) for horizontal speed. Speed error was reduced from 1.49±0.79 ms^{-1} to 0.36±0.09 ms^{-1}, data fusion delivers a four to eight fold improvement in accuracy. (c) Time from triggering the GPS module at the start of a run to receiving a valid position/speed fix, this was almost always (92.8% triggers) within two seconds (d) Time from triggering the GPS module at the start of a run to receiving a high accuracy (horizontal error<10 metres) fix.

69.1% triggers were within two seconds of trigger (e) Time from trigger to receiving high rate (5 Hz) position data 17.2% triggers within two seconds, 52.1% within five seconds and 95.4% within seven seconds. (f) Time from trigger to high rate data against time since last fix (testing the refresh rate required for rapid startup). The substantial delay in high rate data, often of around five seconds, would have a considerable detrimental effect on the accuracy of the fused solution for the start of a run. N=2412 for this figure because startup data are logged for every trigger not just valid runs.

Supplementary Tables

Supplementary Table 1: Information regarding individual dogs in the pack and darting information.

Individual:	Accra	Kigali	Kobe	MJ	Scorpion	Timbuktu
Sex:	Female	Female	Male	Male	Male	Female
Age:	3 years (born 2009)	4 years (born 2008)	3 years (born 2009)	4 years (born 2008)	?	3 years (born 2009)
Rank:	sub-ordinate	sub-ordinate	dominant	sub-ordinate	?	dominant female
Body condition (very poor 1 to excellent 5):	5 (excellent)	4	5 (excellent)	5 (excellent)	5 (excellent)	5 (excellent)
Injury?	No	Healed broken leg (front left) but remaining slight limp and a kiwi sized ball of fluid/blood on the back of the upper leg near the break.	couple of small infection spots (ticks) and slight wound on the top right forelimb	No	No	No
Mass (kg): (empty stomach estimate)	25-26	26	~ 29-30	~ 32	~ 30	~ 29
Hindlimb length (mm): (standing posture)	542	561	528	564	590	528
Forelimb length (mm): (standing posture)	480	476	485	570	490	440
Back length (mm): (top of neck to base of tail)	822	820	880	950	910	810
Drugs:	55 mg xylazine; 50 mg ketamine; 1.2 mg atropine	55 mg xylazine; 50 mg ketamine; 1.2 mg atropine	55 mg xylazine; 50 mg ketamine; 1.1 mg atropine	55 mg xylazine; 50 mg ketamine; 1.1 mg atropine	55 mg zylazine; 50 mg ketamine; 1.1 mg atropine	55 mg xylazine; 50 mg ketamine; 1.2 mg atropine
Time antidote delivered after darting:	46 min; yohimbine 4 mg	51 min; yohimbine 4 mg	45 min; 5.5 mg antisedan	49 min; antisedan 5.5 mg	44 min; yohimbine 4 mg	44 min; yohimbine 4 mg

Supplementary Table 2: Median, lower (Q1) and upper quartile (Q3) and means and standard deviations for, (a) distance travelled per day for focal pack. (b) distance travelled per day other packs. (c) distance between chases, (d) time between chases, (e) number of chases per day and (f) kill rate.

a

	Daily distance travelled (km)	
**Focal pack**	**mean±std**	**median (Q1, Q3)**
total	13.16 ± 7.73	12.51 (7.66 , 17.08)
individual based	13.02 ± 1.12	12.03 (11.53 , 13.21)
Accra	12.87 ± 7.28	12.03 (8.29 , 16.32)
Kigali	14.39 ± 7.70	13.25 (8.34 , 18.62)
Kobe	11.58 ± 8.23	10.70 (5.62 , 16.73)
MJ	12.43 ± 7.79	11.81 (6.26 , 15.99)
Scorpion	13.85 ± 7.56	13.20 (8.58 , 17.69)
Timbuktu*	9.76 ± 7.79	9.35 (2.17 , 14.60)
mean±std of individual medians	11.72 ± 1.50	

c

	Distance between chases (km)	
**Focal pack**	**mean±std**	**median (Q1, Q3)**
total	2.61 ± 2.29	1.90 (1.00,3.47)
individual based	2.57 ± 2.22	1.99 (1.00,3.38)
Accra	2.96 ± 2.57	2.05 (1.05,3.85)
Kigali	2.17 ± 2.06	1.55 (0.82,2.97)
Kobe	2.87 ± 2.72	2.09 (0.78,3.44)
MJ	2.69 ± 2.10	1.94 (1.27,3.47)
Scorpion	2.76 ± 2.28	2.21 (1.20,3.95)
Timbuktu	2.00 ± 1.56	1.40 (0.88,2.62)
mean±std of individual medians	1.87 ± 0.32	

f

**Focal pack**	**Kill rate**
total	0.106
mean±std individual	0.155 ± 0.086
median (Q1,Q3) individual	0.115 (0.1, 0.12)
Accra	0.109
Kigali	0.079
Kobe	0.210
MJ	0.121
Scorpion	0.109
Timbuktu	0.306

* not included due to denning

b

	Daily distance travelled (km)	
**Other packs**	**mean±std**	**median (Q1, Q3)**
total	13.78 ± 8.47	12.77 (7.94 , 18.38)
individual based	14.67 ± 3.91	12.45 (11.42 , 15.35)
Adiga	20.31 ± 9.34	19.28 (14.15 , 26.17)
Augustus	20.54 ± 8.98	21.27 (12.86 , 27.69)
Aztec	16.46 ± 6.79	15.35 (11.97 , 20.95)
Bali	8.94 ± 5.97	8.00 (4.43 , 12.47)
Bobedi	11.75 ± 6.85	11.42 (6.13 , 16.64)
Bongwe	13.95 ± 7.80	12.56 (8.55 , 18.58)
Brian	14.78 ± 7.98	13.93 (9.44 , 16.95)
Dar	11.44 ± 9.05	9.65 (6.63 , 15.21)
Gomer	12.72 ± 7.26	12.06 (8.10 , 17.26)
James	11.54 ± 9.06	10.46 (5.51 , 17.34)
Kubu	12.47 ± 7.99	12.06 (6.19 , 17.52)
Lupe	13.06 ± 6.24	12.34 (8.91 , 16.36)
Pula	17.51 ± 10.58	14.61 (10.08 , 24.45)
Seronera	12.28 ± 6.19	11.95 (8.40 , 15.94)
Stetson	16.56 ± 8.90	14.42 (10.45 , 20.79)
Tangiers	21.03 ± 8.44	20.07 (14.33 , 25.73)
Xerxes	19.82 ± 6.81	18.66 (15.66 , 24.11)
Yolo	8.89 ± 6.88	8.40 (4.28 , 12.44)
mean±std of individual medians	13.69 ± 3.93	

d

	Time between chases (min)	
**Focal pack**	**mean±std**	**median (Q1, Q3)**
total	36.17 ± 39.17	22.06 (11.58,47.33)
individual based	35.62 ± 38.38	22.99 (11.07,45.30)
Accra	40.33 ± 44.38	22.67 (12.48,58.36)
Kigali	30.80 ± 32.62	17.90 (11.06,39.21)
Kobe	40.99 ± 48.02	23.32 (7.09,43.63)
MJ	38.62 ± 40.27	27.47 (13.18,51.01)
Scorpion	36.17 ± 36.56	23.79 (11.81,42.99)
Timbuktu	26.84 ± 28.40	20.33 (10.78,36.62)
mean±std of individual medians	22.58 ± 3.25	

e

	Nr. chases per day	
**Focal pack**	**mean±std**	**median (Q1, Q3)**
total	2.74 ± 1.92	2.00 (1.00 , 4.00)
individual based	2.45 ± 0.88	2.00 (1.00 , 2.00)
Accra	3.02 ± 198	2.00 (1.00 , 4.00)
Kigali	3.16 ± 2.24	4.00 (1.00 , 6.00)
Kobe	0.97 ± 1.78	2.00 (1.00 , 4.00)
MJ	2.68 ± 1.64	2.00 (0.00 , 4.00)
Scorpion	2.33 ± 1.81	1.00 (1.00 , 3.00)
Timbuktu*	1.71 ± 1.44	0.00 (0.00 , 1.00)
mean±std of individual medians	2.43 ± 0.88	

Supplementary Table 3: Median, lower (Q1) and upper quartile (Q3) and means and standard deviations for chase parameters maximum stride speed, chase distance, chase duration, tortuosity, maximum centripetal and tangential accelerations and decelerations, average heading rate and number of strides (a) Median, Q1,Q3 for focal pack (b) Mean and standard deviation for focal pack (c) Median, Q1,Q3 for other packs (d) Mean and standard deviation for other packs (e) Test of difference between focal pack and group of other dogs *p*-values of two sample t-test and Bonferroni corrected *p'*-values. h is 1 if the test rejects the null hypothesis of difference between the samples at the 5% significance level.

a

					median (Q1, Q3)				
Focal pack	max. stride speed (ms^{-1})	chase distance (m)	chase duration (s)	tortuosity (-)	max. centrip. accel. (ms^{-2})	max. centrip. decel. (ms^{-2})	max. tang. accel. (ms^{-2})	max. tang. decel. (ms^{-2})	mean pos. headingrate (deg)
total	10.59 (8.22 , 13.38)	324.19 (170.13 , 599.35)	47.86 (23.39 , 79.47)	1.23 (1.10 , 1.60)	3.58 (2.49 , 4.68)	-3.41 (-4.66 , -2.41)	2.87 (2.05 , 3.86)	-3.33 (-4.45 , -2.24)	12.74 (9.94 , 16.23)
mean individual	10.57 (10.37 , 11.23)	323.76 (315.87 , 348.65)	46.70 (45.64 , 50.12)	1.22 (1.18 , 1.31)	3.46 (3.31 , 3.93)	-3.33 (-3.76 , -3.21)	2.85 (2.73 , 3.07)	-3.39 (-3.46 , -3.16)	12.85 (12.11 , 13.19)
Accra	10.55 (8.11 , 13.07)	348.65 (175.69 , 640.30)	50.12 (22.98 , 83.99)	1.22 (1.08 , 1.50)	3.18 (2.15 , 4.38)	-3.21 (-4.41 , -2.25)	2.77 (1.95 , 3.74)	-3.16 (-4.22 , -2.02)	11.72 (8.85 , 15.98)
Kigali	8.98 (7.51 , 10.96)	315.87 (171.43 , 555.19)	50.56 (25.14 , 83.93)	1.18 (1.09 , 1.51)	3.31 (2.23 , 4.45)	-2.81 (-4.06 , -2.03)	2.69 (1.81 , 3.59)	-2.92 (-3.95 , -1.94)	12.70 (9.90 , 16.75)
Kobe	10.37 (8.41 , 13.66)	322.69 (182.86 , 617.29)	46.21 (25.15 , 70.40)	1.18 (1.09 , 1.59)	3.42 (2.33 , 4.47)	-3.25 (-4.55 , -2.41)	2.73 (2.10 , 4.15)	-3.46 (-4.73 , -2.19)	12.11 (9.27 , 15.05)
MJ	11.23 (8.65 , 13.73)	306.03 (168.97 , 564.57)	42.58 (22.30 , 75.67)	1.21 (1.09 , 1.52)	3.93 (2.85 , 5.14)	-3.76 (-5.15 , -2.66)	3.07 (2.12 , 3.87)	-3.42 (-4.59 , -2.38)	13.20 (10.85 , 16.39)
Scorpion	12.27 (9.88 , 14.78)	360.82 (162.11 , 690.26)	45.64 (20.62 , 78.61)	1.36 (1.13 , 1.76)	4.25 (3.19 , 5.24)	-4.21 (-5.32 , -3.04)	3.15 (2.36 , 4.42)	-3.93 (-5.17 , -2.89)	13.19 (10.51 , 16.66)
Timbuktu	10.58 (8.00 , 12.81)	324.83 (178.25 , 573.17)	47.18 (26.55 , 68.82)	1.31 (1.13 , 1.86)	3.49 (2.53 , 4.37)	-3.41 (-4.33 , -2.74)	2.94 (2.29 , 3.77)	-3.36 (-4.06 , -2.17)	13.01 (10.29 , 16.98)

b

					mean ± std				
Focal pack	max. stride speed (ms^{-1})	chase distance (m)	chase duration (s)	tortuosity (-)	max. centrip. accel. (ms^{-2})	max. centrip. decel. (ms^{-2})	max. tang. accel. (ms^{-2})	max. tang. decel. (ms^{-2})	mean pos. headingrate (deg)
total	10.98 ± 3.37	454.71 ± 402.52	61.88 ± 57.49	5.96E+39 ± 1.98E+41	3.71 ± 1.67	-3.68 ± 1.89	3.05 ± 1.50	-3.51 ± 1.71	14.31 ± 7.10
mean individual	11.08 ± 1.01	447.28 ± 40.16	60.17 ± 5.50	4.01E+39 ± 9.83E+39	3.69 ± 0.39	-3.69 ± 0.41	3.08 ± 0.21	-3.53 ± 0.37	14.40 ± 0.99
Accra	10.77 ± 3.10	479.50 ± 428.76	66.18 ± 58.95	1.79 ± 2.10	3.37 ± 1.56	-3.47 ± 1.74	2.85 ± 1.38	-3.28 ± 1.63	13.30 ± 6.95
Kigali	9.51 ± 2.70	424.68 ± 387.92	67.63 ± 70.25	2.41E+40 ± 3.99E+40	3.46 ± 1.57	-3.20 ± 2.11	2.88 ± 1.50	-3.12 ± 1.58	14.09 ± 6.27
Kobe	11.28 ± 3.55	422.24 ± 313.16	54.85 ± 38.25	1.78 ± 1.97	3.53 ± 1.86	-3.52 ± 1.57	3.16 ± 1.60	-3.50 ± 1.66	13.32 ± 6.51
MJ	11.43 ± 3.30	451.24 ± 412.60	58.59 ± 55.20	2.22 ± 6.05	3.98 ± 1.65	-3.96 ± 1.78	3.09 ± 1.43	-3.61 ± 1.66	14.78 ± 6.80
Scorpion	12.60 ± 3.68	506.88 ± 445.29	58.77 ± 51.17	2.28 ± 3.80	4.35 ± 1.75	-4.35 ± 1.90	3.42 ± 1.68	-4.20 ± 1.87	15.31 ± 7.90
Timbuktu	10.87 ± 3.21	399.11 ± 304.21	55.02 ± 42.27	1.96 ± 1.80	3.48 ± 1.36	-3.63 ± 1.54	3.05 ± 1.25	-3.47 ± 1.66	15.60 ± 9.28

c

					median (Q1, Q3)				
Other packs	max. stride speed (ms^{-1})	chase distance (m)	chase duration (s)	tortuosity (-)	max. centrip. accel. (ms^{-2})	max. centrip. decel. (ms^{-2})	max. tang. accel. (ms^{-2})	max. tang. decel. (ms^{-2})	mean pos. headingrate (deg)
total	12.51 (10.11 , 14.80)	431.35 (220.20 , 549.97)	51.82 (28.68 , 59.30)	1.19 (1.07 , 1.60)	4.10 (2.92 , 5.42)	-3.73 (-5.16 , -2.72)	3.30 (2.42 , 4.19)	-3.21 (-4.36 , -2.36)	11.55 (9.19 , 14.63)
mean individual	12.15 (11.02 , 13.07)	403.71 (331.20 , 431.30)	47.03 (37.37 , 53.89)	1.13 (1.12 , 1.18)	3.76 (3.42 , 4.14)	-3.82 (-4.78 , -3.16)	3.23 (2.88 , 3.38)	-3.21 (-3.44 , -2.95)	11.23 (10.73 , 12.21)
Bobedi	13.14 (10.53 , 15.18)	447.83 (240.65 , 602.87)	57.76 (29.39 , 60.59)	1.23 (1.09 , 1.82)	4.32 (3.19 , 5.48)	-4.05 (-5.19 , -2.80)	3.43 (2.49 , 4.27)	-3.36 (-4.50 , -2.39)	11.55 (9.72 , 14.74)
Brian	13.01 (10.94 , 15.40)	269.74 (147.34 , 512.61)	30.69 (20.01 , 55.02)	1.14 (1.07 , 1.35)	3.55 (3.26 , 4.77)	-5.51 (-5.99 , -3.55)	3.33 (2.79 , 4.42)	-3.53 (-4.37 , -2.39)	12.86 (10.94 , 15.14)
Gomer	10.76 (9.16 , 11.71)	414.77 (295.76 , 471.22)	50.01 (36.95 , 57.19)	1.11 (1.04 , 1.38)	3.96 (2.45 , 5.33)	-3.58 (-4.57 , -2.37)	2.62 (2.28 , 4.06)	-2.84 (-3.95 , -2.34)	10.56 (7.55 , 13.44)
James	11.29 (9.60 , 13.94)	392.66 (205.35 , 456.01)	44.05 (28.66 , 55.73)	1.12 (1.05 , 1.27)	3.29 (2.41 , 4.28)	-2.75 (-4.83 , -1.35)	3.14 (1.93 , 4.02)	-3.06 (-4.01 , -2.09)	10.90 (7.72 , 14.65)

d

					mean ± std				
Other packs	max. stride speed (ms^{-1})	chase distance (m)	chase duration (s)	tortuosity (-)	max. centrip. accel. (ms^{-2})	max. centrip. decel. (ms^{-2})	max. tang. accel. (ms^{-2})	max. tang. decel. (ms^{-2})	mean pos. headingrate (deg)
total	12.54 ± 3.29	427.26 ± 248.33	48.62 ± 23.41	1.52 ± 0.87	4.14 ± 1.85	-3.96 ± 1.81	3.44 ± 1.68	-3.42 ± 1.60	12.29 ± 4.74
mean individual	12.27 ± 0.94	376.52 ± 62.58	44.19 ± 6.90	1.35 ± 0.22	3.90 ± 0.36	-3.96 ± 0.92	3.37 ± 0.30	-3.34 ± 0.21	12.35 ± 1.08
Bobedi	12.94 ± 3.55	467.90 ± 271.82	51.44 ± 24.64	1.67 ± 1.03	4.35 ± 1.81	-4.14 ± 1.80	3.56 ± 1.73	-3.53 ± 1.72	12.41 ± 4.46
Brian	13.09 ± 2.43	327.88 ± 215.59	35.65 ± 19.19	1.27 ± 0.33	3.80 ± 1.78	-5.15 ± 1.71	3.64 ± 1.80	-3.51 ± 1.21	13.46 ± 3.72
Gomer	11.05 ± 2.13	362.88 ± 146.71	47.68 ± 20.37	1.25 ± 0.30	3.97 ± 2.24	-3.58 ± 1.50	3.29 ± 1.74	-3.17 ± 1.45	10.87 ± 3.73
James	12.02 ± 3.10	347.45 ± 197.68	41.98 ± 20.54	1.20 ± 0.21	3.48 ± 1.53	-2.97 ± 1.84	2.98 ± 1.34	-3.16 ± 1.35	12.67 ± 7.01

e

Comparing focal pack to group of 4 other individuals using two sided t-test and bonferroni correction for medians									
p value	0.152115468	0.773284595	0.058620184	0.518031101	0.339300401	0.198680602	0.422122755	0.048181142	<0.001
h	0	0	0	0	0	0	0	1	1
p'	0.912692807	1.036062203	0.410341291	1.266368264	1.357201604	0.993403009	1.357201604	0.385449132	<0.001
h'	0	0	0	0	0	0	0	0	1
